# Seasonal migration patterns and the maintenance of evolutionary diversity in a cryptic bird radiation

**DOI:** 10.1111/mec.16241

**Published:** 2021-11-05

**Authors:** Qindong Tang, Reto Burri, Yang Liu, Alexander Suh, Gombobaatar Sundev, Gerald Heckel, Manuel Schweizer

**Affiliations:** ^1^ Institute of Ecology and Evolution University of Bern Bern Switzerland; ^2^ Natural History Museum Bern Switzerland; ^3^ Schweizerische Vogelwarte Sempach Switzerland; ^4^ State Key Laboratory of Biocontrol College of Ecology School of Life Science Sun Yat‐sen University Guangzhou China; ^5^ School of Biological Sciences—Organisms and the Environment University of East Anglia Norwich UK; ^6^ Department of Organismal Biology – Systematic Biology, Evolutionary Biology Centre (EBC) Uppsala University Uppsala Sweden; ^7^ National University of Mongolia and Mongolian Ornithological Society Ulaanbaatar Mongolia

**Keywords:** allochrony, cryptic diversification, phylogeography, population genomics, *Riparia diluta*, Sand martin

## Abstract

Morphological differentiation associated with evolutionary diversification is often explained with adaptive benefits but the processes and mechanisms maintaining cryptic diversity are still poorly understood. Using genome‐wide data, we show here that the pale sand martin *Riparia diluta* in Central and East Asia consists of three genetically deeply differentiated lineages which vary only gradually in morphology but broadly reflect traditional taxonomy. We detected no signs of gene flow along the eastern edge of the Qinghai‐Tibetan plateau between lowland south‐eastern Chinese *R. d*. *fohkienensis* and high‐altitude *R. d*. *tibetana*. Largely different breeding and migration timing between these low and high altitude populations as indicated by phenology data suggests that allochrony might act as prezygotic isolation mechanism in the area where their ranges abut. Mongolian populations of *R. d*. *tibetana*, however, displayed signs of limited mixed ancestries with Central Asian *R. d*. *diluta*. Their ranges meet in the area of a well‐known avian migratory divide, where western lineages take a western migration route around the Qinghai‐Tibetan plateau to winter quarters in South Asia, and eastern lineages take an eastern route to Southeast Asia. This might also be the case between western *R. d*. *diluta* and eastern *R. d*. *tibetana* as indicated by differing wintering grounds. We hypothesize that hybrids might have nonoptimal intermediate migration routes and selection against them might restrict gene flow. Although further potential isolation mechanisms might exist in the pale sand martin, our study points towards contrasting migration behaviour as an important factor in maintaining evolutionary diversity under morphological stasis.

## 1 INTRODUCTION

How biodiversity is generated and maintained remains one of the main questions in evolutionary biology. Studies on the evolution of ecological and phenotypic diversity within rapidly multiplying lineages ‐ often adaptive radiations ‐ have been instrumental for our understanding on how adaptive processes trigger speciation (McGee et al., 2020; Nosil, 2012; Schluter, 2000; Yoder et al., 2010). However, divergent adaptation is not the only path towards speciation and evolutionary diversification does not necessarily result in remarkable morphological and ecological differentiation. In so‐called nonadaptive radiations (Rundell & Price, 2009), lineages diversify within similar environments in allopatry or parapatry, and thus under similar regimes of natural selection. Such radiations can therefore be accompanied by minimal morphological and ecological differentiation (e.g., Fink et al., 2010). As a result, this may lead to genetically differentiated, yet morphologically cryptic lineages but differences in other aspects such as sexual signalling, physiology or phenology might have evolved (Braune et al., 2008; Feckler et al., 2014; Taylor & Friesen, 2017).

Over the last two decades, an increasing number of such morphologically cryptic lineages has been discovered through genetic methods, thus uncovering an unexpected evolutionary diversity across the tree of life (e.g., Kozak et al., 2006; Leys et al., 2017; Slavenko et al., 2020; Weir et al., 2016). Indeed, a considerable proportion of biodiversity may be constituted of “cryptic species”, that is, evolutionary lineages with restricted gene flow that “do not form diagnostic morphological clusters” (Struck et al., 2018). Investigating their evolution is important to determine the processes under which biodiversity evolves in general and to increase our still limited understanding of diversification under morphological stasis in particular (Fišer et al., 2018). The latter may contrast with the processes and mechanisms governing adaptive radiations, for example, in intensively studied systems such as Darwin's finches (Grant & Grant, 2011) or African lake cichlids (McGee et al., 2020; Seehausen, 2006).

Unlike the seemingly rare cases of explosive diversification in adaptive radiations, speciation is generally considered a slow process with spatial isolation usually required to initiate lineage divergence (Price, 2008; Tobias et al., 2020). After an initial phase of allopatry during the speciation process, the critical question is whether or not the differentiated lineages withstand gene flow at secondary contact. In the context of cryptic radiations, investigating evolutionary processes that might restrict gene flow among lineages lacking obvious morphological differences is crucial for our understanding on how diversity can be maintained in such cases (e.g., Beysard & Heckel, 2014; Beysard et al., 2015).

The breakdown of lineage integrity – often referred to as “speciation reversal” (Kearns et al., 2018; Seehausen et al., 2008) – is the expected outcome of secondary contact in taxa with insufficient or ephemeral reproductive isolation. Extensive hybridization between morphologically cryptic lineages and the potentially resulting fusion of lineages, however, might remain difficult to detect, unless species complexes are sampled comprehensively across their ranges and analysed with genome‐wide data (Slager et al., 2020). However, morphologically cryptic lineages might have different adaptations in life‐history traits not reflected in morphology. These might include contrasting habitat preferences precluding secondary sympatry or different timing of reproduction facilitating co‐existence through temporal segregation (allochrony) (Leys et al., 2017; Taylor & Friesen, 2017). Additionally, morphologically cryptic lineages might have diverged in social signals (Tobias et al., 2020) and thus show premating isolation at secondary contact. The resulting assortative mating by itself might not be sufficient to prevent eventual lineage fusion, unless hybrid fitness is reduced (Irwin, 2020). Such postzygotic isolation might not only be caused by hybrids showing intermediate or “transgressive” signals, but also by genetic incompatibilities. The evolution of genetic incompatibilities, however, is usually considered to be too slow to generally play an important role at the onset of the speciation process (Price, 2008; Price & Bouvier, 2002), although the relationship between divergence time in allopatry and different stages along the speciation continuum remains poorly understood (Beysard & Heckel, 2014; Dufresnes et al., 2019). Interestingly, in recent studies on cryptic sister species of birds in Amazonia, strong postzygotic reproductive isolation was found with little evidence for premating isolation even in relatively young pairs (Cronemberger et al., 2020; Pulido‐Santacruz et al., 2018). Whether genetic incompatibilities among cryptic species might play a more important role than previously thought and actually accumulate faster than premating isolation under certain circumstances (Cronemberger et al., 2020) is under debate, and remains to be tested in additional geographic contexts and systems.

**FIGURE 1:**
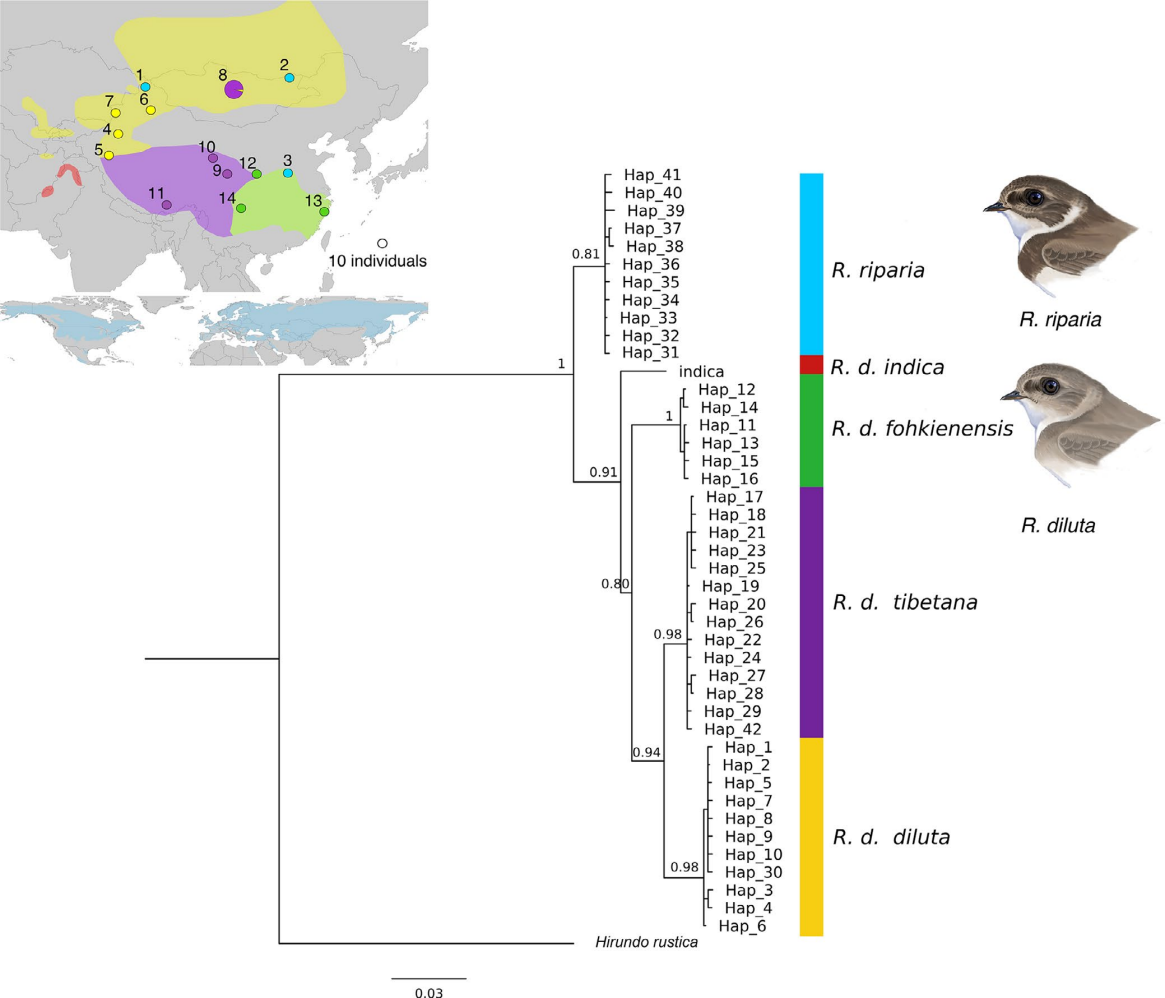
Bayesian majority rule consensus tree based on haplotypes of the mtDNA gene NADH dehydrogenase subunit II (ND2) of *R*. *diluta* and *R*. *riparia*. Bayesian posterior probabilities are given for major nodes. Colour shades indicate the potential breeding ranges of different subspecies of *R*. *diluta* (top) and of *R*. *riparia* (bottom) modified from Bird Life International and Handbook of the Birds of the World (2016). Sampled populations are shown with dots on the maps with colours corresponding to mtDNA clades in the phylogenetic tree. *R*. *diluta* samples collected in Mongolia clustered with *R. d*. *tibetana* except one individual that was found in the clade of *R. d*. *diluta*. Paintings by Manuel Schweizer

The processes and mechanisms that lead to morphologically cryptic divergence and maintain genetic partitioning thereafter are best amenable to research in systems where multiple cryptic lineages are found in geographic contact. The pale sand martin (*Riparia diluta*) of Central and East Asia provides such a promising natural system to study diversification under morphological stasis. Its four recognized subspecies overlap in morphometrics and have no diagnostic differences in plumage features (Schweizer et al., 2018). The subtle differences in plumage comprise color shade, prominence of ear‐covert coloration as well as extension of breast‐band, but the identification of single individuals without context is usually not possible (Schweizer et al., 2018; Shirihai & Svensson, 2018). This crypsis in morphology contrasts with deep phylogeographic structure in mtDNA among subspecies (Schweizer et al., 2018). For a long time, these birds were considered conspecific with the collared sand martin *Riparia riparia* that has a Holarctic distribution. The ranges of *R*. *diluta* and *R*. *riparia* widely overlap in East Asia without apparent interbreeding, and they show subtle but consistent differences in plumage features, vocalizations and genetics (Gavrilov & Savchenko, 1991; Goroshko, 1993; Pavlova et al., 2008; Schweizer & Aye, 2007). The morphologically cryptic phylogeographic lineages within *R*. *diluta* occur in different climate zones and widely differing altitudinal ranges. Nominate *R. d*. *diluta* is found in the steppes of Central Asia, *R. d*. *fohkienensis* in subtropical south China, *R. d*. *indica* in the north‐western part of the Indian subcontinent, and *R. d*. *tibetana* breeds on the Qinghai‐Tibetan plateau (Figure 1). While *R. d*. *indica* is geographically isolated, the breeding areas of the remaining subspecies are thought to be largely contiguous, but geographic sampling in previous studies was limited, especially in areas of potential secondary contact (Schweizer et al., 2018).

Here, analysing variation at >7100 single nucleotide polymorphisms (SNPs) based on a comprehensive geographic sampling of pale sand martin populations around the Qinghai‐Tibetan plateau, we aimed to test (a) if the deep phylogeographic structure indicated by mtDNA is also reflected in genome‐wide variation and (b) if the phylogeographic lineages indeed overlap in morphospace and can thus be considered cryptic. Finally, we (c) investigated, if gene flow between phylogeographic lineages is actually reduced in areas of potential contact.

## 2 MATERIALS AND METHODS

### 2.1 Sampling and DNA extraction

Blood was collected from 149 *Riparia* individuals (119 *R*. *diluta*; 30 *R*. *riparia*) on their breeding grounds (Figure 1, Table S1). We sampled 14 breeding colonies of *R*. *diluta* covering the breeding areas of three subspecies and including potential areas of contact among them at the north‐western and eastern edges of the Qinghai‐Tibetan plateau, and additionally three colonies of *R*. *riparia*. This included 37 individuals from four populations of *R. d*. *diluta* from north‐western China, 29 individuals from three populations of *R. d*. *tibetana* from the Qinghai‐Tibetan plateau, 29 individuals from three populations of *R. d*. *fohkienensis* from south‐eastern China, and 24 individuals of *R. d*. *diluta* from Mongolia. We grouped three pairs of breeding colonies from the same geographic regions in one population each resulting in a total of 14 populations (11 for *R*. *diluta* and 3 for *R*. *riparia*) for further analyses (Table S1, Figure 1). All samples were preserved in 99% ethanol and later stored at −20°C. DNA was extracted with a modified salt extraction protocol (Aljanabi & Martinez, 1997). Birds were captured and sampled under permits and approvals from the relevant authorities in China and Mongolia.

### 2.2 Sequencing and analysis of mtDNA

To investigate whether the deep phylogeographic structure among subspecies in mtDNA can be recovered with a more extensive geographic sampling, we amplified a fragment (~850 bp) of the mitochondrial NADH dehydrogenase subunit 2 (ND2) gene of all 149 individuals using the same protocol as Schweizer et al. (2018). Sanger sequencing was performed in both directions by LGC Genomics GmbH (Germany). Complementary strands were aligned using the package SeqMan in DNAstar (Burland, 2000). Unique ND2 haplotypes were detected using dnasp V6.12 (Rozas et al., 2017). Phylogenetic reconstruction based on unique haplotypes was done using maximum likelihood (ML) and Bayesian inference (BI). Barn swallow *Hirundo rustica* (GeneBank accession number DQ176515) was used as outgroup and one sequence of *R. d*. *indica* (Schweizer et al., 2018; GeneBank accession number MG881167) was additionally added. Sequence alignment was done using ClustalW in mega 7 (Kumar et al., 2016). TN93+I was selected as the best‐fitting model of nucleotide substitution with phymltest in PhyML (Guindon & Gascuel, 2003) with r 3.6.2 (R core Team, 2013). ML tree search was employed in PhyML with 1000 bootstrap replicates. BI was performed with mrbayes 3.2.7 (Ronquist et al., 2012) with four independent runs of Metropolis‐coupled Markov chain Monte Carlo analyses. Each run comprised one cold chain and three heated chains at a default temperature of 0.1. The chains were run for 20 million generations and sampled every 100 generations. tracer 1.7 (Rambaut et al., 2018) was used to assess the length of burn‐in and to confirm adequate effective sample sizes (ESS > 200) of the posterior distribution. Clades were considered as supported by our analyses when bootstrap values were >70% (Hillis & Bull, 1993) and clade credibility values for the BI > 0.95 (Huelsenbeck & Ronquist, 2001). The final ML and BI phylogenetic trees were edited for display in figtree v1.4.4 (http://tree.bio.ed.ac.uk/software/figtree/).

### 2.3 De novo reference genome sequencing and assembly

A draft genome was sequenced and assembled de novo by National Genomic Infrastructure Stockholm, Sweden, from a male *R*. *riparia* collected in Zhengzhou, Henan province, China in 2017 (SYSb6505, Table S2). DNA extraction and linked‐read sequencing were performed as in Lutgen et al. (2020). In brief, DNA was extracted using the Qiagen MagAttract HMW DNA kit following the manufacturer's instructions except for using the double volume and prolonging the digestion time. A single linked‐read sequencing library was then prepared using the 10× Genomics Chromium Genome library kit, and sequenced on half an S4 lane on a NovaSeq 6000 instrument at NGI Stockholm. A de novo reference genome was assembled with supernova assembler version 2.1.0 (Weisenfeld et al., 2017). We obtained a pseudohaploid draft reference with a total assembly length of 1.18 Gb, effective read coverage of 40.9x and scaffold N50 of 12.6 Mb. All scaffolds were then mapped to the well annotated genome of zebra finch *Taeniopygia guttata* version bTaeGut1_v1.p (Korlach et al., 2017) using minimap2 (Li, 2018). Only scaffolds that were larger than 1000 bp and uniquely mapped to the zebra finch reference genome were retained with the largest scaffold being 32.8 Mb in length. As the reference genome was from a male (homomorphic sex ZZ in birds), scaffolds mapping to the Z chromosome were excluded in further analyses to avoid underestimation of heterozygosity in females (heteromorphic sex ZW). Moreover, scaffolds of the mitochondrial genome were also excluded and thus only autosomal SNPs retained. The total length of the final assembly was 980 Mb. Genome heterozygosity as the proportion of heterozygous sites of the sand martin reference genome was estimated based on k‐mer count distribution of kmer length of 21 (*m* = 21) using Jellyfish (Marçais & Kingsford, 2011) combined with GenomeScope (http://qb.cshl.edu/genomescope/) (Vurture et al., 2017).

### 2.4 Genotyping by sequencing

Genotyping by sequencing (GBS) (Elshire et al., 2011) was conducted by ecogenics GmbH (Switzerland). Individually MID‐tagged reduced representation libraries were generated using the standard enzyme combination EcoRI/Msel and single‐end reads of 75 bp were sequenced on an Illumina NextSeq instrument. After checking the quality of the raw reads with fastqc version 0.10.1 (Andrew, 2019), leading and trailing low quality reads were removed using trimmomatic version 0.39 (Bolger et al., 2014). Trimmed reads were aligned to the collared sand martin draft genome using bwa mem version 0.7.17 (Li, 2013). Single‐nucleotide polymorphisms (SNPs) were called and genotyped with angsd (Korneliussen et al., 2014) based on GATK genotype likelihoods (McKenna et al., 2010) retaining sites with *p* < .001 for being variable, a minimum mapping quality of 20, a minimum base quality score of 20, a minimum total read depth of 300, a minimum individual read depth of five and minor allele frequency of 0.05 (‐GL 2 ‐SNP_pval 1e‐3 ‐minQ 20 ‐minMapQ 20 ‐setMinDepth 300 ‐geno_minDepth 5 ‐minMaf 0.05). We only retained uniquely mapped reads and biallelic SNPs with <10% missing data (‐uniqueOnly 1 ‐skipTriallelic 1 ‐minInd 135).

### 2.5 Analyses of population genomic structure

To examine genetic structure among populations, we first conducted a principal component analysis (PCA) based on individual genotype likelihoods using PCAngsd (Meisner & Albrechtsen, 2018). This was done for *R*. *diluta* and *R*. *riparia* together and for *R*. *diluta* separately. The eigenvectors from the covariance matrix were computed using the function “eigen” in r 3.6.2. To check for potential gene flow between populations, particularly in putative contact zones, we also performed admixture analysis in NGSadmix (Skotte et al., 2013). NGSadmix was also based on genotype‐likelihood, thus accounting for the uncertainty of called genotypes. It was run ten times on all individuals (*R*. *diluta* and *R*. *riparia* together) for each K and the number of ancestral populations K set from 2 to 10. The optimal K was evaluated using clumpak (Kopelman et al., 2015).

We additionally did two analyses of molecular variance (AMOVA) and computed pairwise *F*_ST_ between populations of *R*. *diluta* in arlequin version 3.5.2.2 (Excoffier & Lischer, 2010) using SNPs with less than 5% missing data (default setting) and 1000 permutations. For AMOVA, the three subspecies were defined as groups with the Mongolian population either included in *R. d*. *tibetana* or in *R. d*. *diluta*. The distance matrix was computed using pairwise distances. The input file for arlequin was generated using pgdspider version 2.1.1.5 (Lischer & Excoffier, 2012). To investigate the influence of geographical distance on population structure, we tested for isolation by distance between different populations of *R*. *diluta*. We applied Mantel tests (Mantel, 1967) to half matrices of genetic (*F*_ST_/(1‐*F*_ST_)) and logarithmic (ln) Euclidean geographic distances between populations using the r package ade4 (Dray & Dufour, 2007) with 999 Monte‐Carlo permutations.

Nucleotide diversity (π) of each population was computed using angsd (Korneliussen et al., 2014). Filters for SNPs were again set to a minimum mapping quality of 20, a minimum base quality score of 20, minimum read depth of five for each individual, and less than 10% missing data. We only kept uniquely mapped reads for the estimation of posterior probabilities of sample allele frequency (SAF) for each population, and then computed the site frequency spectrum (SFS) using realSFS. Pairwise nucleotide diversity for each site was computed using thetaStat based on the SFS and the average was used as nucleotide diversity for each population. The unfolded SFS was estimated using the reference genome of *R*. *riparia* for the characterization of ancestral states in populations of *R*. *diluta*. For populations or *R*. *riparia*, the folded SFS was used.

### 2.6 Morphological analysis

To investigate the extent of morphological differences between subspecies of *R*. *diluta*, we collected mensural data of 190 individuals. Eighty‐four of these were also included in the GBS analyses (complete data could not be obtained for the remaining 35 individuals used for GBS), subspecific identity of the others was based on breeding colony origin. Eight traits were measured following Eck et al. (2012): length of bill tip to feathering, bill depth, bill width, wing length, length of P8 (third outermost primary), tail length, length of tail fork and tarsus length (Table S3). In an additional analysis, we combined our data set with the one of Schweizer et al. (2018) from which three morphometric traits (wing length, tail length and length of tail fork) for 120 individuals of *R*. *diluta* (32 of *R. d*. *diluta*, 19 of *R. d*. *tibetana*, 36 of *R. d*. *indica*, 33 of *R. d*. *fohkienensis*) were available stemming mainly from museum specimens. After log‐transformation of measurements, morphometric differences among subspecies were explored using a principal component analysis (PCA) on the correlation matrix using the function prcomp of the r package stats.

### 2.7 Seasonal occurrence patterns

As *R*. *diluta* occurs in different climate zones and across a broad altitudinal range, we assessed differences in seasonal occurrence patterns (phenology) between different geographic regions corresponding to the supposed distribution of the three subspecies. To this end, we used data from our own fieldwork and compiled records of *R*. *diluta* from the two citizen science databases ebird (https://ebird.org/home) and BirdReport of China (http://www.birdrecord.cn/). Each record was allocated to three periods in each month, that is, before the 10th, between the 10th and the 20th, and after the 20th. The three geographic regions were defined as follows: (a) south and central China east of the Qinghai‐Tibetan plateau below 3000 m above sea level (asl) corresponding to the supposed breeding distribution of *R. d*. *fohkienensis*, (b) Qinghai‐Tibetan plateau above 3000 m asl corresponding to the supposed breeding range of *R. d*. *tibetana*, (c) the region of China north‐west of the Qinghai‐Tibetan plateau corresponding to the supposed breeding distribution of *R. d*. *diluta*.

## 3 RESULTS

### 3.1 MtDNA phylogeny

The final ND2 alignment was 851 bp in length with 32 haplotypes in *R*. *diluta* and 11 haplotypes in *R*. *riparia*. Within *R*. *diluta*, BI and ML phylogenetic reconstructions recovered well supported clades mostly consistent with the distribution ranges of the morphologically‐defined subspecies (Figure 1 and Figure S1): One clade consisted of all samples of *R. d*. *fohkienensis* from lowland south‐eastern China, one of *R. d*. *tibetana* from the Qinghai‐Tibetan Plateau and one of *R. d*. *diluta* from north‐western China (Figure 1). Samples collected from Mongolia in the potential breeding range of *R. d*. *diluta*, however, clustered and shared haplotypes with samples of *R. d*. *tibetana* from the Qinghai‐Tibetan plateau. Only one individual from Mongolia with a unique haplotype (Hap 30) did not cluster with the remaining samples from this region and *R. d*. *tibetana*, and was instead found in the *R. d*. *diluta* clade. The positions of these different clades as well as that of *R. d*. *indica* were not robustly supported throughout. The monophyly of the haplotypes found in *R*. *riparia* was only supported in ML analyses, but not with BI.

### 3.2 Population genomics of nuclear variation

**FIGURE 2:**
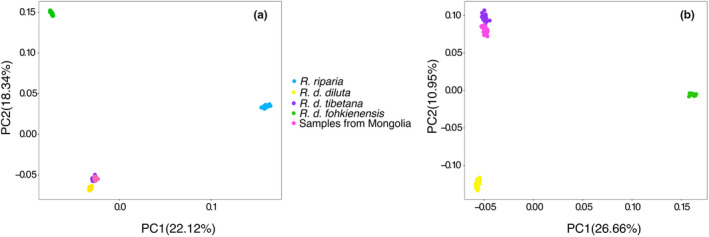
Principal component analysis (PCA) on nuclear SNPs of *R*. *diluta* and *R*. *riparia* (a, based on 7,640 SNPs) and only of *R*. *diluta* (b, based on 7118 SNPs). Colours correspond to different subspecies except pink dots indicate the samples collected from Mongolia in the potential breeding range of *R. d*. *diluta*

The heterozygosity of the collared sand martin reference genome was estimated to be 0.1%. In total, we obtained 7640 autosomal SNPs in the data set including *R*. *diluta* and *R*. *riparia* after aligning trimmed GBS reads to the collared sand martin draft genome. These SNPs were distributed across most autosomes except for the two microchromosomes 16 (1.22 Mb) and 29 (4.21 Mb), and there was a highly significant positive correlation (*r* = .92; *p* = 5.02 × 10^−14^) between the number of SNPs called per chromosome and chromosome size (Figures S2 and S3). In a PCA on both species, *R*. *diluta* was clearly separated from *R*. *riparia* in PC1 (22.12% of the variance), while PC2 (18.34% of the variance) separated *R. d*. *fohkienensis* and the remaining samples of *R*. *diluta* (Figure 2a). It is noteworthy that all *R*. *diluta* samples from Mongolia clustered with *R. d*. *tibetana*. In a separate PCA with 7,118 autosomal SNPs called for the 119 *R*. *diluta* samples only (Figure 2b), *R. d*. *fohkienensis* was clearly differentiated from the remaining samples along PC1 (26.66% of variance). PC2 (10.95% of variance) separated north‐western Chinese *R. d*. *diluta* from a cluster containing *R*. *tibetana* from the Qinghai‐Tibetan plateau and all samples from Mongolia.

**FIGURE 3:**
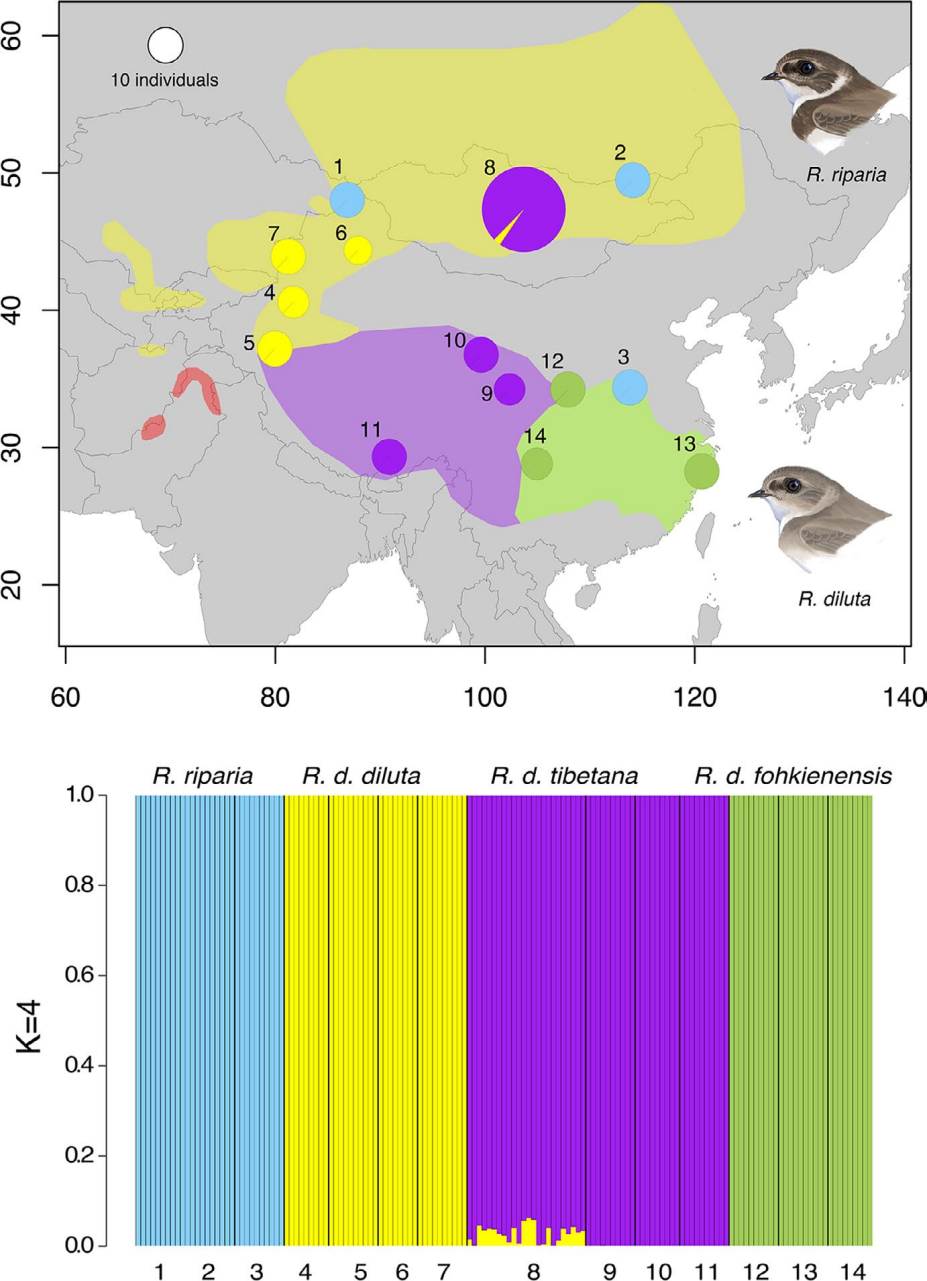
Top: Map with pie charts representing genome‐wide ancestry assignment in *Riparia* populations with *K* = 4 using NgsAdmix. Limited admixture between *R. d*. *diluta* and *R. d*. *tibetana* was found in the population from Mongolia (8) with *K* = 4. Colour shades indicate the potential breeding ranges of different subspecies of *R*. *diluta*. Pie sizes are proportional to sample sizes. Bottom: bar plots showing individual ancestry assignments with *K* = 4. Numbers correspond to the different populations on the map. No individuals from the range of *R. d*. *indica* indicated in red on the map could be sampled

Admixture analyses resulted in *K* = 4 as the best‐fitting number of ancestral populations with a somewhat lower likelihood for *K* = 3 (Figure S4). However, *R*. *riparia*, *R. d*. *fohkienensis* and north‐western Chinese *R. d*. *diluta* were always resolved as distinct genetic clusters with separate ancestries (Figure 3 and Figure S5). For *K* = 4, *R. d*. *tibetana* from the Qinghai‐Tibetan plateau and individuals from Mongolia formed one additional genetic cluster, with the latter showing limited evidence of mixed ancestry with north‐western Chinese *R. d*. *diluta* (average 2.9%; range per individual: 0 to 6.2%).

Alternative AMOVAs and F‐statistics based on 6857 SNPs supported the closer affinity of Mongolian birds to *R. d*. *tibetana*. Overall differentiation between populations was very high with *F*_ST_ = 0.225 (*p* < .0001). When the Mongolian population was grouped in an AMOVA with *R. d*. *tibetana*, the total variation explained by the subspecies reached *F*_CT_ = 0.216 with very little differentiation within these groups (*F*_SC_ = 0.011, both *p* < .0001). When the Mongolian population was alternatively grouped with *R. d*. *diluta*, the proportion of the explained variation dropped to *F*_CT_ = 0.186 and differentiation within the groups of populations increased accordingly (*F*_SC_ = 0.045; both *p*‐values < .0001). We thus consider the population in Mongolia to belong to *R. d*. *tibetana* outside the assumed distribution range of this subspecies. With this assignment, pairwise *F*_ST_ between populations within subspecies ranged from 0 to 0.051 (most *p* < .05; Table S4) while all pairwise comparisons between populations from different subspecies ranged between *F*_ST_ = 0.1 and 0.36 (all *p* < .05; Table S4).

**FIGURE 4:**
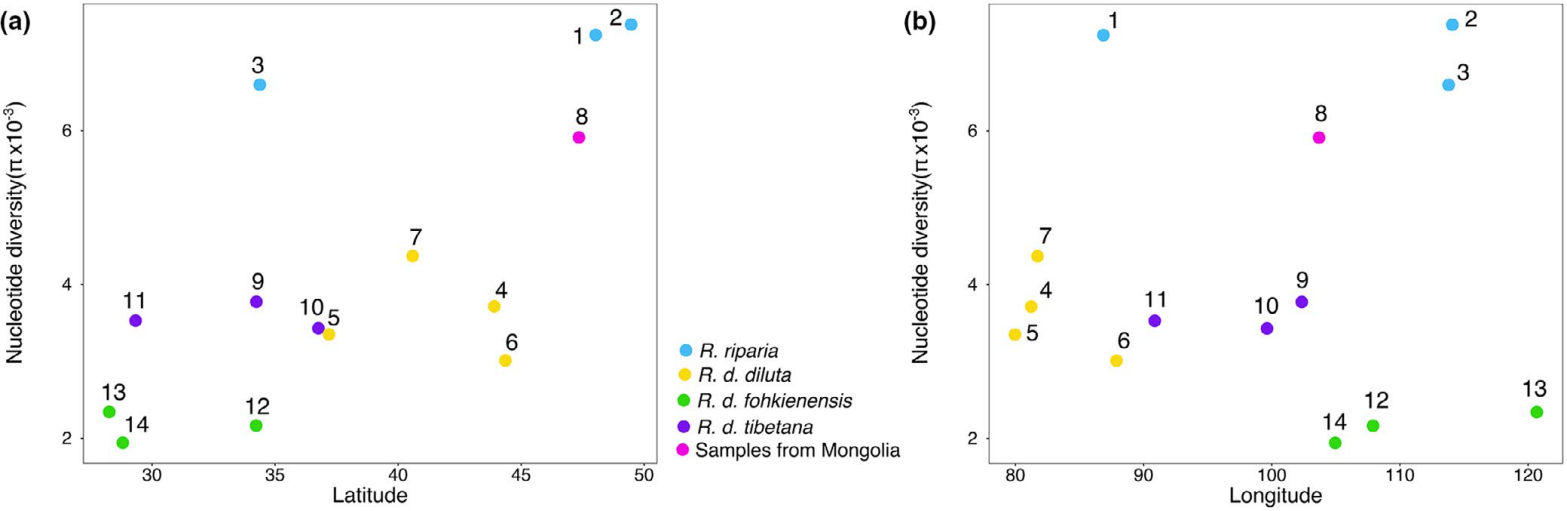
Nucleotide diversity (π) of *R*. *diluta* and *R*. *riparia* populations plotted against latitude (a) and longitude (b). Numbers correspond to the different populations shown in Figures 1 and 3

Comparison of molecular diversity showed further that the Mongolian birds featured the highest nucleotide diversity among all analysed *R*. *diluta* populations (π = 5.9 × 10^−3^). Nucleotide diversity was overall similar across *R. d*. *diluta* and *R. d*. *tibetana* populations from the Qinghai‐Tibetan plateau with no detectable relation to longitude or latitude (π ranging from 3.09 × 10^−3^ to 4.49 × 10^−3^; Figure 4). All populations of *R. d*. *fohkienensis* showed comparatively lower levels of nucleotide diversity (π ranging from 1.99 × 10^−3^ to 2.39 × 10^−3^). The highest values of nucleotide diversity were found in the three analysed populations of *R*. *riparia* (π ranging from 6.69 × 10^−3^ to 7.49 × 10^−3^).

### 3.3 Isolation by distance

**FIGURE 5:**
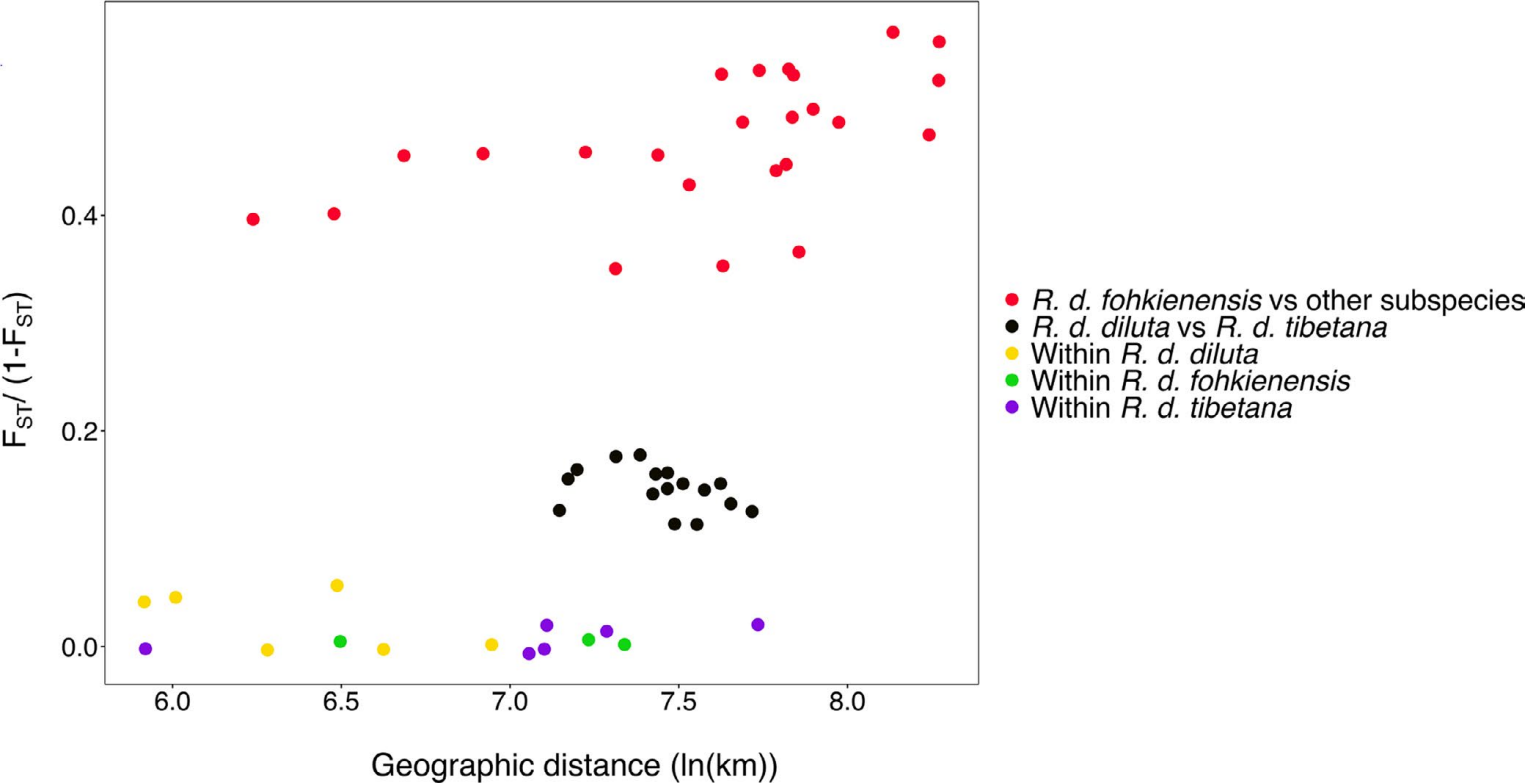
Genetic differentiation between populations of *R*. *diluta* belonging to three subspecies relative to geographic distance. Genetic differentiation between populations of different subspecies was considerably larger than comparisons within subspecies

Given distances of thousands of kilometres between the analysed populations, we tested for the importance of isolation by distance in genetic differentiation between and within subspecies of *R*. *diluta*. Overall, a Mantel test detected a highly significant relationship between genetic differentiation and spatial distance between populations (*r* = .5486; *p* = .001; Figure 5). Closer inspection revealed that this relationship was mainly driven by very high pairwise comparisons between subspecies across their parapatric distribution ranges. In contrast, comparisons among large spatial distances within subspecies, especially in *R. d*. *tibetana*, showed no evidence of elevated genetic differentiation (Figure 5). Accordingly, a Mantel test restricted to pairwise comparisons within subspecies provided no evidence of isolation by distance (*r* = −.304; *p* = .87), indicating considerable dispersal among populations over large distances.

### 3.4 Morphological differentiation

**FIGURE 6:**
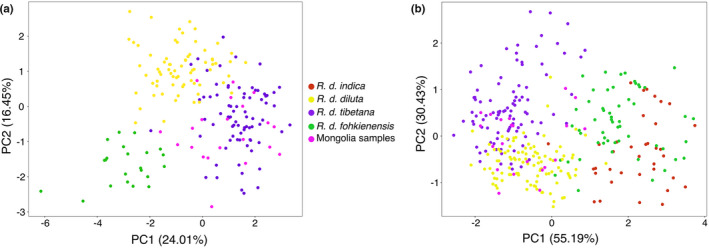
Principal component analysis (PCA) of subspecies within *R*. *diluta* based on eight (a), and on three morphometric traits (b) with additional samples from a previous analysis (Schweizer et al., 2018) and including *R. d*. *indica*. Colours correspond to the different subspecies, except pink dots which indicate samples collected in Mongolia

A PCA on eight morphological traits measured on the novel *R*. *diluta* samples presented here revealed gradual differences between subspecies with some overlap (Figure 6a). PC1 (24.01% variance) separated largely *R. d*. *fohkienensis* from *R. d*. *tibetana* and was mainly influenced by length of P8, wing length and tail length (loading factors of 0.507, 0.498 and 0.452, respectively, Table S5) while PC 2 mainly distinguished *R. d*. *fohkienensis* from *R. d*. *diluta* and was dominated by the length of tail fork (loading factor of 0.668, Table S5). Birds from Mongolia clustered among individuals of *R*. *tibetana* with similarly gradual transitions to the other subspecies (Figure 6a).

The analysis of three morphological traits only, which enabled the inclusion of additional 120 individuals (including *R. d*. *indica*) from Schweizer et al. (2018), revealed overall less separation between different subspecies of *R*. *diluta* (Figure 6b). PC1 (55.19% of variance) separated *R. d*. *fohkienensis* and *R. d*. *indica* together from *R. d*. *diluta* and *R. d*. *tibetana* in PC1), while PC2 (30.43% of variance) tended to differentiate subspecies of these two pairs. However, there was gradual overlap overall.

### 3.5 Differences in phenology

**FIGURE 7:**
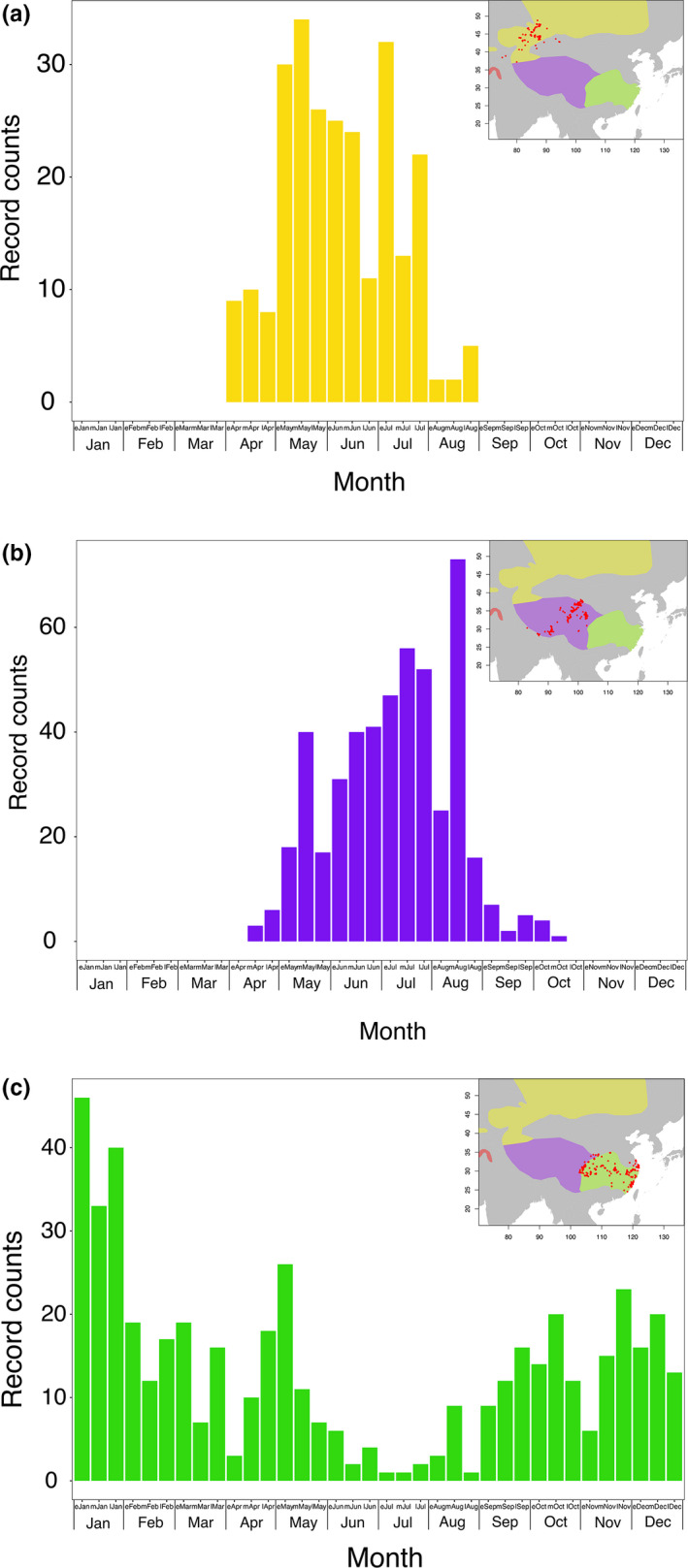
Phenology of 1253 records of *R*. *diluta* in China between 1985 and 2021. (a) Records in the breeding range of *R. d*. *diluta* to the north‐west of the Qinghai‐Tibetan plateau. (b) Records in the breeding range of *R. d*. *tibetana* on the Qinghai‐Tibetan plateau above 3,000 m above sea level (asl). (c) Records in the breeding range of *R. d*. *fohkienensis* in south and central China east of the Qinghai‐Tibetan plateau below 3000 m asl. Colour shades in the maps indicate the potential breeding ranges of different subspecies of *R*. *diluta* (yellow: *R. d*. *diluta*, purple: *R. d*. *tibetana*, green: *R. d*. *fohkienensis*). Each record is shown as a red dot on the maps for the respective geographic areas

A total of 1253 records (545 from BirdReport of China and 708 from ebird) of pale sand martin from China were compiled for the years 1985–2021. Pale sand martins were found throughout the year but with distinct differences between the regions occupied by the three subspecies. In north‐western China in the breeding area of *R. d*. *diluta*, the first birds appeared in late April, peaks were revealed in May and July, and there were no records from September onwards (Figure 7a). A similar pattern of occurrence was revealed on the Qinghai‐Tibetan plateau above 3000 m asl in the breeding range of *R. d*. *tibetana* (Figure 7b). The first birds were recorded in mid‐April with a broad peak of records around mid‐July and mid‐August and no records after mid‐October. In south and central China below 3000 m asl however, records were found throughout the year with most records of pale sand martin stemming from the winter months with a reduction during the summer months (Figure 7c). Although this area corresponds to the traditional breeding range of *R. d*. *fohkienensis*, migrant and wintering individuals of other subspecies are certainly included in these records. According to our field observations, *R. d*. *fohkienensis* in the area was breeding already in late April with chicks found in holes until late May (c. Table S1) while *R. d*. *tibetana* on the Qinghai‐Tibetan plateau started to build nest‐holes only in the beginning of June.

## 4 DISCUSSION

Here, by using population genomic data, we show that the pale sand martin *Riparia diluta* contains multiple deep evolutionary lineages despite extremely subtle and gradual morphological variation among them and little genetic differences within lineages over large geographic areas. Differentiation patterns in nuclear SNPs are largely consistent with phylogeographic structure in mtDNA (see also Schweizer et al., 2018) and represent different levels. We discuss how the integrity of the evolutionary lineages could be maintained despite morphological stasis. We hypothesize that prezygotic isolation in terms of allochrony and extrinsic postzygotic isolation caused by contrasting migration directions might prevent lineage fusion.

### 4.1 Phylogeographic structure

Evolutionary lineages within pale sand martin *Riparia diluta* are largely consistent with geographical distribution and at least partly with taxonomic classification of subspecies. *R. d*. *fohkienensis* has diverged most, and there were no signs of admixture between less divergent *R. d*. *tibetana* and *R. d*. *diluta* in the region of presumed contact at the western edge of the Qinghai‐Tibetan plateau. Individuals of *R. d*. *tibetana* in Mongolia however, displayed signs of limited mixed ancestries with *R. d*. *diluta* from north‐western China indicating the absence of full reproductive isolation between these subspecies and potentially recent admixture.

The strong differentiation in genetically distinct but morphologically cryptic lineages within *R*. *diluta* is in stark contrast with the lack of any phylogeographic structure in its sister species, the collared sand martin *R*. *riparia*, in our study region. Geographically widespread nuclear homogeneity in *R*. *riparia* is in agreement with shallow mtDNA diversity over its entire Holarctic breeding range indicating recent demographic expansion (Pavlova et al., 2008; Schweizer et al., 2018). In contrast, extensive genetic structure in *R*. *diluta* is more similar to other Central and East Asian bird species complexes and has been related to heterogeneous environments and/or a climate being only mildly affected by Pleistocene climate changes that enabled the persistence of isolated populations in mountainous region at the south‐western edge of the Qinghai‐Tibetan plateau (e.g., Qu et al., 2014; Liu et al., 2016, 2020). Varying nucleotide diversity among different populations of *R*. *diluta* indicates contrasting recent demographic histories, which need to be further investigated.

The distribution of *R. d*. *tibetana* is usually considered to be restricted to the Qinghai‐Tibetan plateau (e.g., del Hoyo & Collar, 2016), but we showed here with genome‐wide data that this evolutionary lineage extends into central Mongolia. This is consistent with cases of shared mtDNA haplotypes between birds from Mongolia and Russia with *R. d*. *tibetana* from the Qinghai‐Tibetan plateau (Schweizer et al., 2018). It remains to be examined how much the range of *R. d*. *tibetana* extends farther to the north and west from central Mongolia.

### 4.2 Cryptic diversification with different levels of reproductive isolation

We detected no evidence of gene flow in the potential areas of contact between lowland *R. d*. *fohkienensis* and *R. d*. *tibetana* from the Qinghai‐Tibetan plateau. Allochrony – differences in the timing of breeding ‐ in combination with ecological divergence may play an important role in the prevention of hybridization between them. Data on seasonal occurrence patterns and our own observations suggest that breeding of *R. d*. *fohkienensis* in the lowlands of central and south China takes place considerably earlier than that of *R. d*. *tibetana* on the Qinghai‐Tibetan plateau. Hence, their different phenologies ‐ probably connected to differing migration behaviour with *R. d*. *fohkienensis* probably only conducting short‐distance movements unlike *R. d*. *tibetana* (see below) ‐ might act as prezygotic isolation mechanisms. Speciation through allochrony, that is, temporal segregation of breeding populations as an important contributor to reproductive isolation, has been invoked in several animal groups, including birds (e.g., Bearhop et al., 2005; Friesen et al., 2007; Gómez‐Bahamón et al., 2020; Kimmitt et al., 2019; Sirkiä et al., 2018; Taylor et al., 2018, 2019; Taylor & Friesen, 2017). However, other potential prezygotic isolation mechanisms such as differences in mating behaviour between *R. d*. *fohkienensis* and *R. d*. *tibetana* remain to be investigated. Moreover, the involvement of additional factors cannot be excluded, especially ecological differences as *R. d*. *fohkienensis* and *R. d*. *tibetana* might have adapted to different climate regimes. Comparatively fast rates of climate‐niche evolution could be expected in the temporally and spatially heterogeneous climate in the region of the Qinghai‐Tibetan plateau and central and south China (c. Lawson & Weir, 2014).

However, we cannot exclude the existence of an undetected contact zone with hybridization between *R. d*. *fohkienensis* and *R. d*. *tibetana* at the edge of the plateau given that the closest sampled populations were 512 km apart. Hybrid zones between bird species may be considerably narrower, but in bird taxa with recent divergence, relatively wide hybrid zones of >100 km have also been found (Price, 2008). Hybrid zone width is, among other factors, strongly influenced by dispersal distance (Barton & Hewitt, 1985; McEntee et al., 2020). This has not been studied in *R*. *diluta*, rendering it difficult to make predictions about expected potential hybrid zone width. In *R*. *riparia*, its sister species, however, 7% of juveniles were found >199 km away from their natal colonies in consecutive years in Britain (Mead, 1979) indicating comparatively high colonisation potential (Tittler et al., 2009). Considerable dispersal between colonies over large distances is also indicated in *R*. *diluta* by low genetic differentiation and a lack of patterns of isolation by distance within evolutionary lineages. Finer‐scale sampling of the potential contact area would be necessary to specifically test for interbreeding. Given the absence of any traces of admixture in the sampled populations (e.g. Figure 3), large‐scale gene flow between *R. d*. *fohkienensis* and *R. d*. *tibetana* has probably ceased comparatively long ago given that divergence of *R. d*. *fohkienensis* has been estimated to have occurred about 1.2 million years ago (Schweizer et al., 2018).

Our results clearly show closer relationships between *R. d*. *diluta* and *R. d*. *tibetana* and thus the possibility of hybridization between them may not be too unexpected. It is probably more surprising that the signals of limited autosomal introgression were not detected at the north‐western edge of the Qinghai Tibetan plateau (cf. Figure 1), but rather into Mongolian *R. d*. *tibetana*. The comparatively high levels of nucleotide diversity in the Mongolian population of *R. d*. *tibetana* might also be a consequence of admixture with *R. d*. *diluta*. Further sampling in western Mongolia would be needed to determine the extent and spatial structure of hybridization between the two.

The mountains of Central Mongolia between north‐western Chinese populations of *R. d*. *diluta* and Mongolian *R. d*. *tibetana* have been identified as potentially limiting extensive hybridization between two subspecies of barn swallow *Hirundo rustica* which show contrasting migration routes around the Qinghai‐Tibetan plateau (Scordato et al., 2020). The area where the lineages of *R. d*. *diluta* and *R. d*. *tibetana* might be in contact in Mongolia is indeed located in a migratory divide in different bird species complexes (Irwin & Irwin, 2005; Scordato et al., 2020). The Qinghai‐Tibetan plateau has been proposed as a major barrier to bird migration and a majority of migrant Siberian species use just one migratory route – east or west – around it or show different routes in different subspecies (Irwin & Irwin, 2005). *R. d*. *diluta* has been recorded to winter in the north‐western part of the Indian Subcontinent and rarely on the Arabian peninsula, and probably takes a western route around the Qinghai‐Tibetan plateau with migration documented in north‐western South Asia (Rasmussen et al., 2005; Shirihai & Svensson, 2018). By contrast, *R. d*. *tibetana* might winter in Southeast Asia and circumnavigate the Qinghai‐Tibetan plateau in the east or follow river valleys at its south‐eastern edge and winter in the northern, central and north‐eastern Indian Subcontinent (own data; Rasmussen et al., 2005). As shown for several songbird species, migration direction may have a genetic basis and thus hybrids may have nonoptimal intermediate migration routes and might be selected against (Berthold et al., 1992; Delmore, Hübner, et al., 2015; Delmore, Kenyon, et al., 2015; Delmore & Irwin, 2014; Helbig, 1991, 1996; Lundberg et al., 2017). In combination with geographical barriers, such a mechanism might prevent lineage fusion between *R. d*. *diluta* and *R. d*. *tibetana* in Mongolia. Secondary contact of populations with different migration directions could trigger the evolution of prezygotic isolation mechanisms (Scordato et al., 2020). Given the morphological crypsis of the lineages in *R*. *diluta*, we hypothesize that the secondary contact might be too recent and/or admixture not extensive enough for this process to play a role. However, mating behaviour of the evolutionary lineages remains to be thoroughly investigated to reveal potentially hitherto undocumented differences.

### 4.3 Conclusion

Genome‐wide differentiation between the morphologically cryptic lineages of the pale sand martin *Riparia diluta* represents different levels, and we hypothesize that they might differ in their strength of reproductive isolation. While genomic differentiation of *R. d*. *fohkienensis* suggests effective reproductive isolation for a comparatively long time period, footprints of introgression were found between *R. d*. *diluta* and *R. d*. *tibetana*. In the absence of obvious sexually selected traits, seasonal migration behaviour might be an essential factor in maintaining genetic integrity of these morphologically cryptic evolutionary lineages.

Seasonal migration behaviour has for a long time been considered as playing an important role in generating and maintaining evolutionary divergence (reviewed in Turbek et al., 2018). It has been hypothesized that fusion of different evolutionary lineages might be prevented by contrasting migration behavior even with comparatively little differentiation in other traits (Delmore, Hübner, et al., 2015; Delmore, Kenyon, et al., 2015). Here, we add an additional aspect to this: seasonal migration behaviour might be an essential mechanism to maintain evolutionary diversity under morphological stasis.

## AUTHOR CONTRIBUTIONS

Manuel Schweizer and Gerald Heckel conceived the study; Qindong Tang, Manuel Schweizer and Gombobaatar Sundev collected samples with assistance from Yang Liu. Qindong Tang conducted laboratory work and analysed the data together with Gerald Heckel and Manuel Schweizer and assistance from Reto Burri; Alexander Suh and Reto Burri contributed materials; Qindong Tang, Manuel Schweizer and Gerald Heckel wrote the manuscript with support from all authors.

## Supporting information

Figure S1‐S5

Table S1‐S5

## Data Availability

The data supporting this study have been made openly available on GenBank under accessions MZ747656–MZ747697 for *ND2* haplotypes, as well as on the NCBI sequence read archive (SRA) under BioProject PRJNA755835 with accession nos. SRR15558367–SRR15558515 and BioSample numbers SAMN20856160–SAMN20856308 for raw individual GBS sequences, and accession no. JAIXNV000000000 and BioSample no. SAMN20845799 and for the genome assembly.
