# Supplementary material for: Seasonal migration patterns and the maintenance of evolutionary diversity in a cryptic bird radiation
Source: Mol Ecol. 2021 Nov 5;31(2):632–45. doi: 10.1111/mec.16241 (PMC9298432; doi:10.1111/mec.16241)
Supplement: Supplementary file 1 — Figure S1‐S5 [file MEC-31-632-s002.docx]

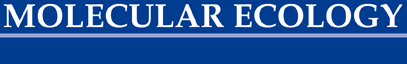


**Supplemental Information for:**

**Seasonal migration patterns and the maintenance of evolutionary diversity in a cryptic bird radiation**

Qindong Tang, Reto Burri, Yang Liu, Alexander Suh, Gombobaatar Sundev, Gerald Heckel Manuel Schweizer

**Table of Contents:**

| **Supplementary of Figure S1:** Maximum  likelihood phylogeny based on ND2 haplotypes. | Page 1 |
| --- | --- |
| **Supplementary of Figure S2:** Number of SNPs  genotyped in *Riparia diluta* and *R. riparia*  populations per autosome. | Page 2 |
| **Supplementary of Figure S3:** Correlation  between the number of SNPs genotyped  per autosome and chromosome size. | Page 3 |
| **Supplementary of Figure S4:** ΔK statistics for  admixture analysis from K=2 to K=10. | Page 4 |
| **Supplementary of Figure S5:** Individual  ancestry assignment for admixture analysis from  K=2 to K=10. | Page 5 |


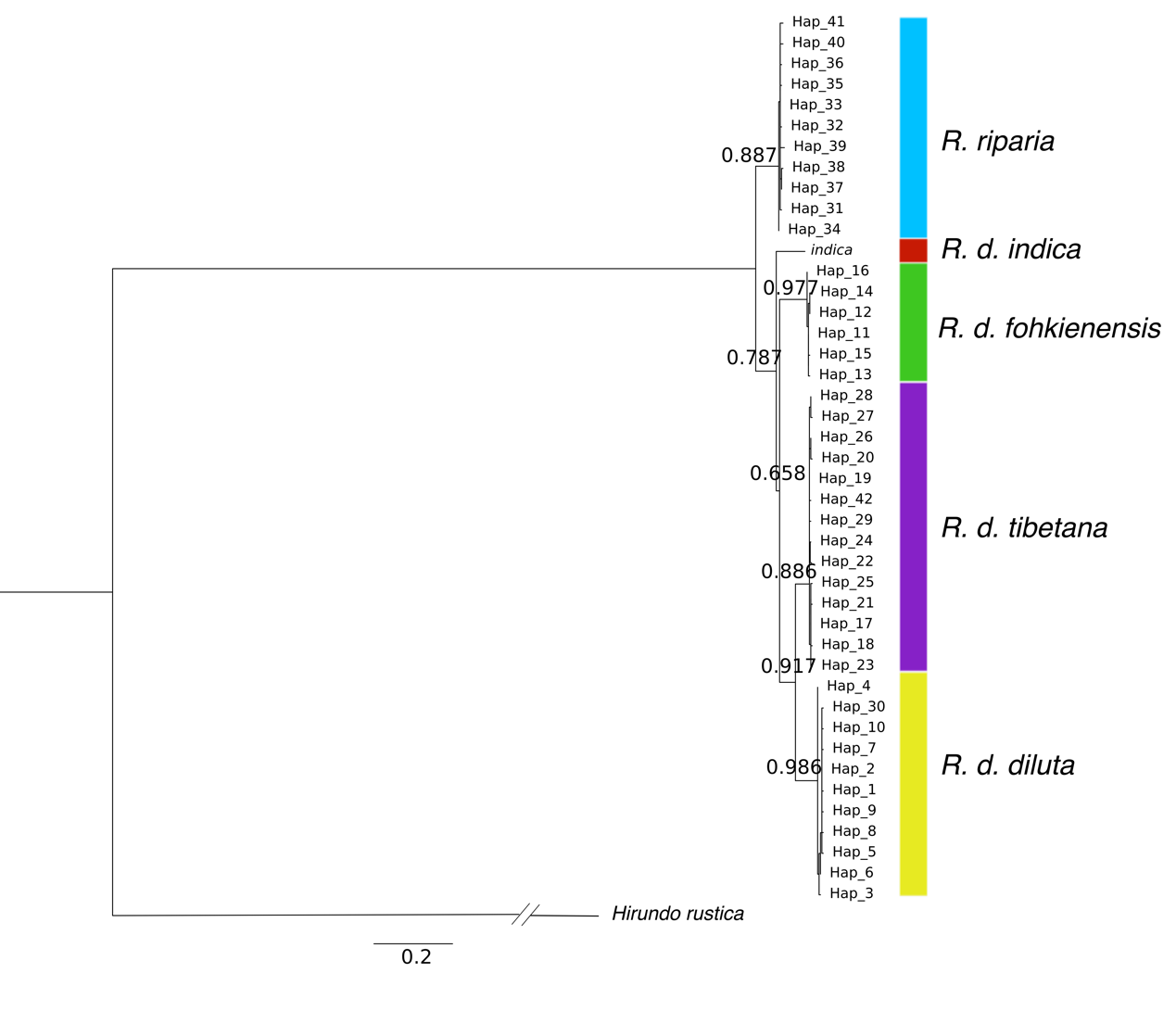


Figure S1. Maximum likelihood phylogeny based on haplotypes of the mtDNA gene NADH dehydrogenase subunit II (ND2) of *R. diluta* and *R. riparia*. Bootstrap values are given for major nodes.


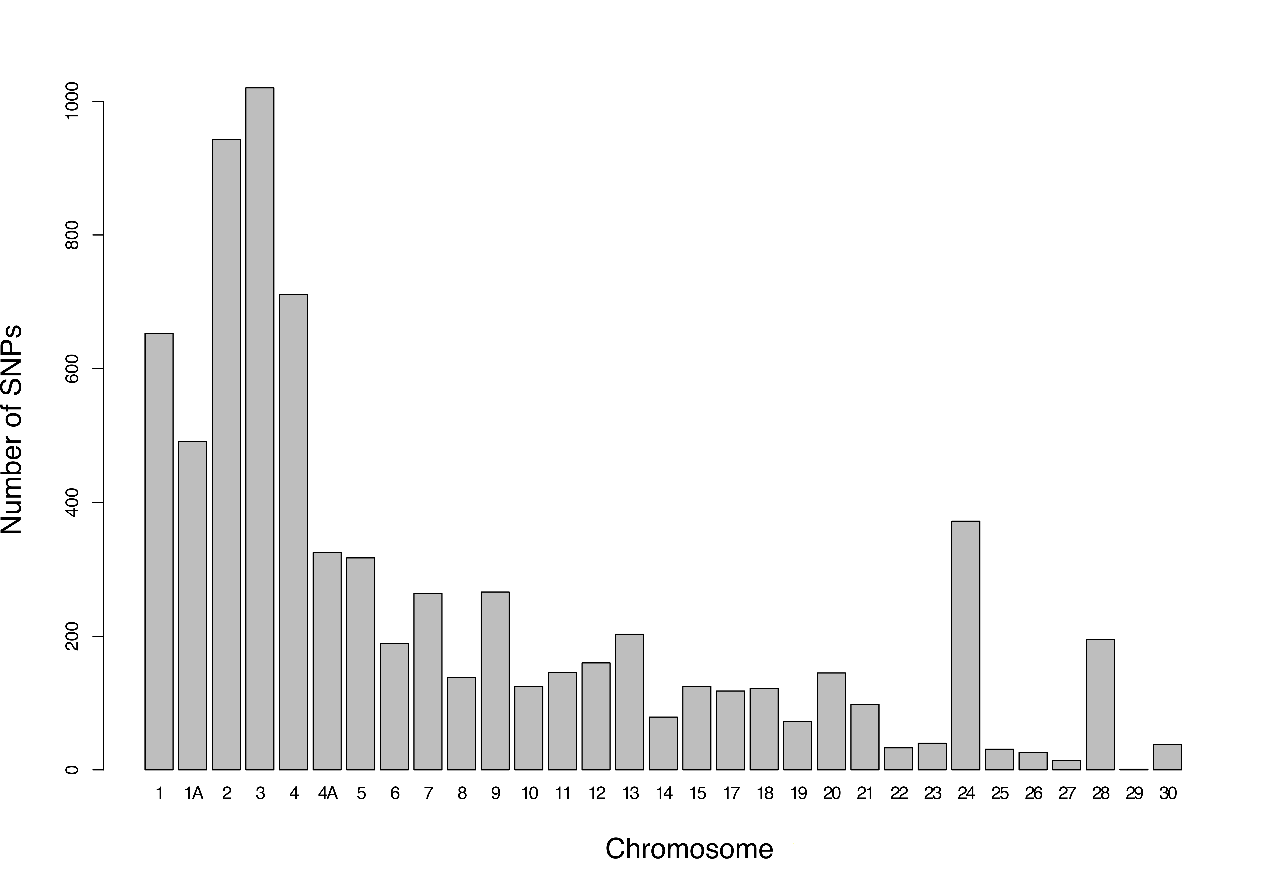


Figure S2. Number of SNPs genotyped in *Riparia diluta* and *R. riparia* populations per autosome.


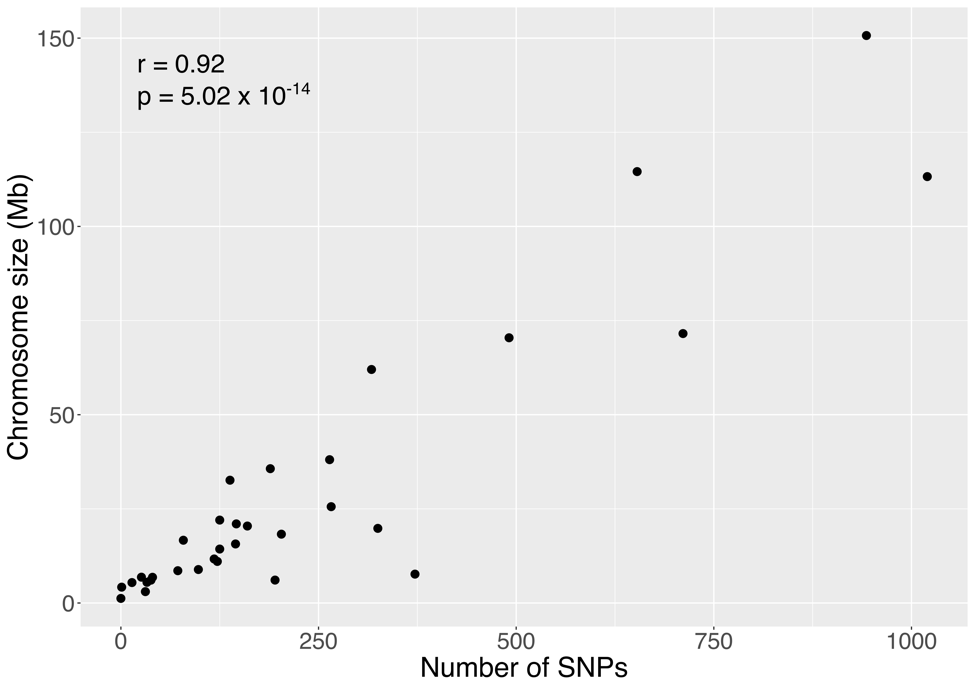


Figure S3. Relationship between the number of SNPs genotyped per autosome and chromosome size in megabases (Mb).


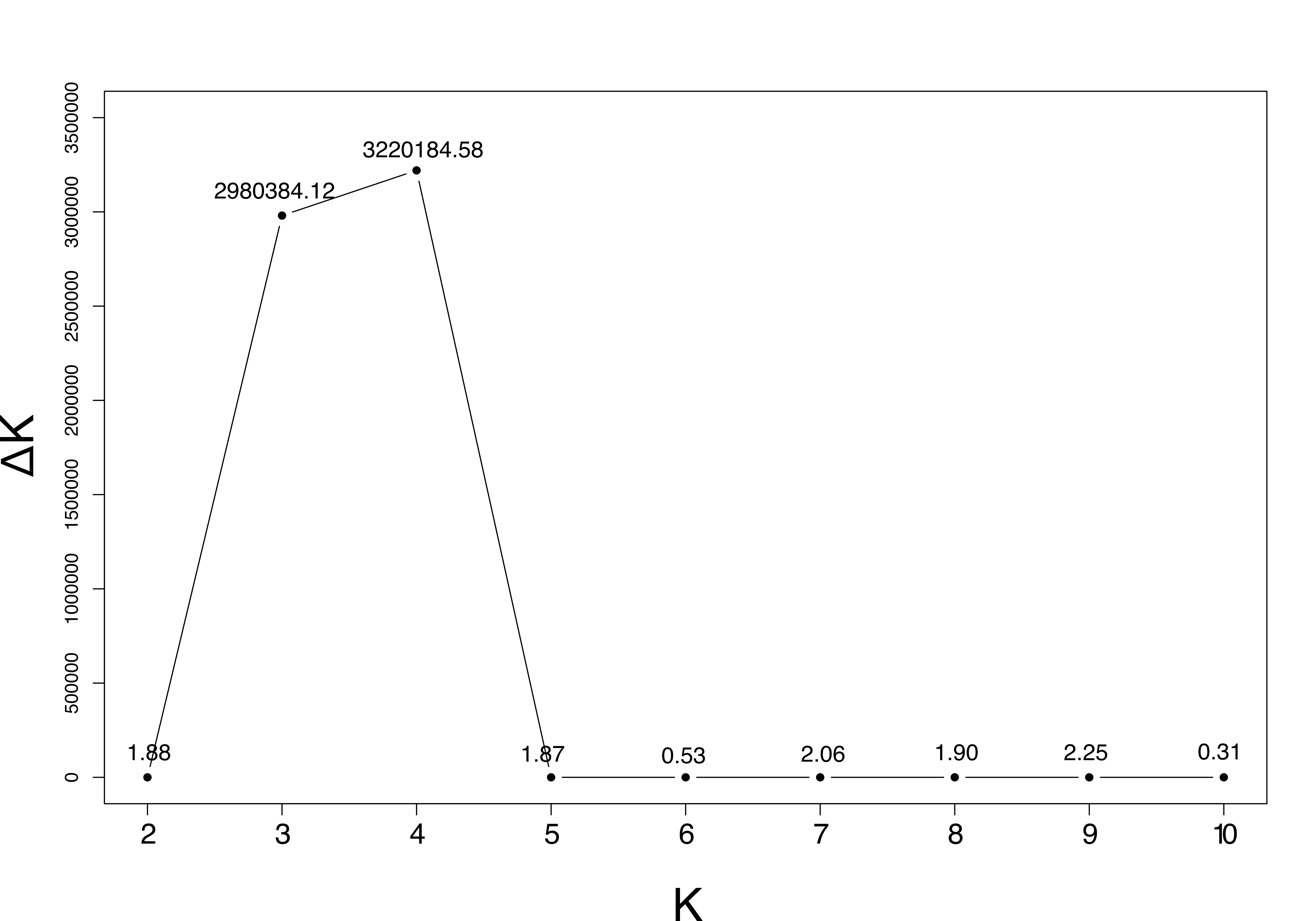


Figure S4. ΔK statistics for admixture analysis from K=2 to K=10.


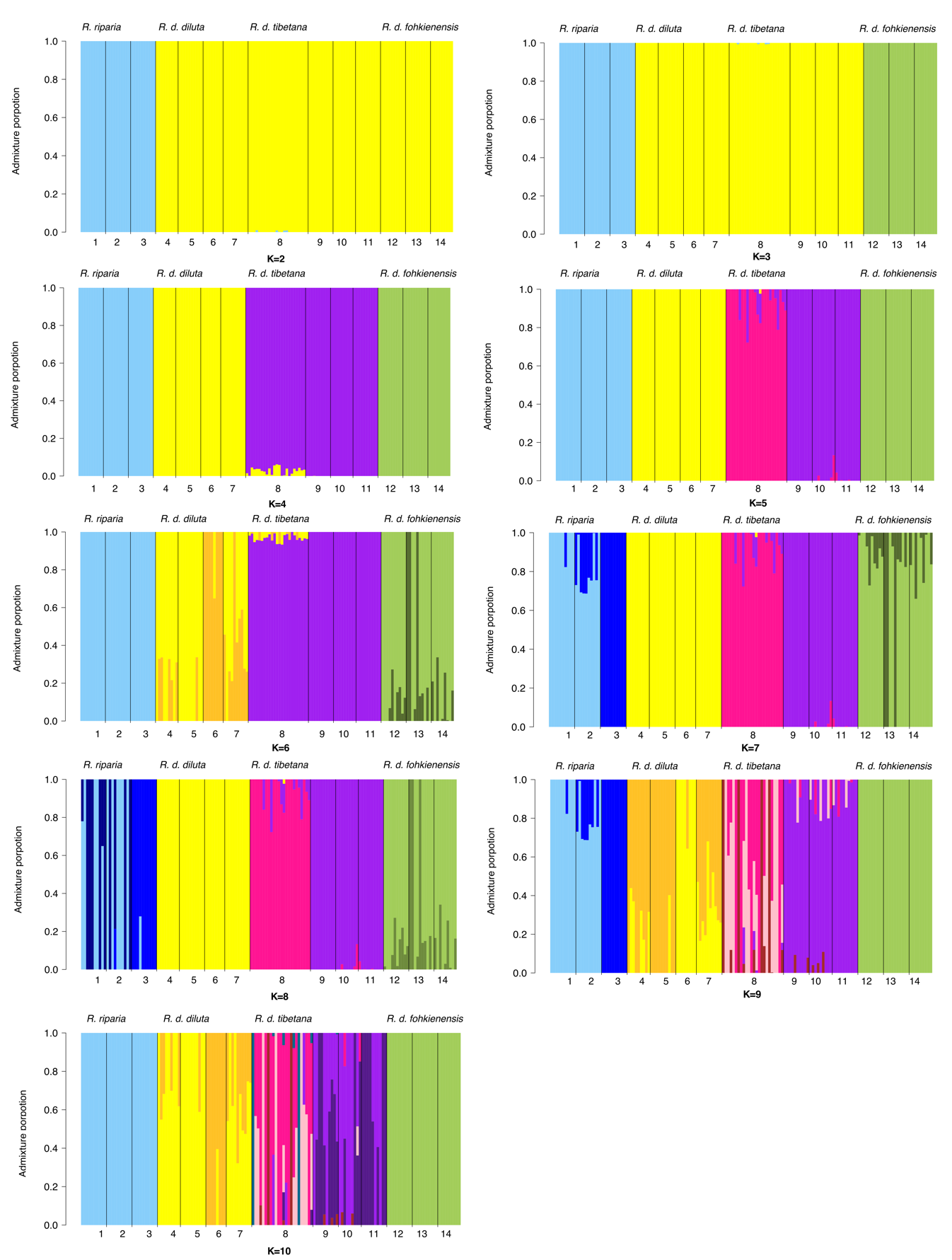


Figure S5. Individual ancestry assignment for admixture analysis from from K=2 to K=10. Numbers correspond to population IDs shown in Figure 3. Taxon labels for different populations were based on mitochondrial lineages.
