# Supplementary material for: Seasonal migration patterns and the maintenance of evolutionary diversity in a cryptic bird radiation
Source: Mol Ecol. 2021 Nov 5;31(2):632–45. doi: 10.1111/mec.16241 (PMC9298432; doi:10.1111/mec.16241)
Supplement: Supplementary file 2 — Table S1‐S5 [file MEC-31-632-s001.docx]

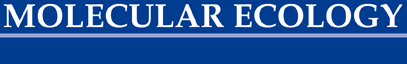


**Supplemental Information for:**

**Seasonal migration patterns and the maintenance of evolutionary diversity in a cryptic bird radiation**

Qindong Tang, Reto Burri, Yang Liu, Alexander Suh, Gombobaatar Sundev, Gerald Heckel Manuel Schweizer

**Table of Contents:**

| **Supplementary of Table S1:** Population information. | Page 1 |
| --- | --- |
| **Supplementary of Table S2:** Sample list and  accession numbers for each individual. | Page 2 |
| **Supplementary of Table S3:** Morphological data. | Page 9 |
| **Supplementary of Table S4:** Pairwise F_ST_ and  geographic distances between populations. | Page 17 |
| **Supplementary of Table S5:** Factor loadings of  principal component analysis on  morphometric measurements. | Page 18 |

Table S1 Population information. We grouped breeding colonies 8a and 8b, 9a and 9b, 10a and 10b as three populations (8, 9, 10 in Figure 3) separately since they were breeding in the same geographic region and we selected the coordinates of the first colony for analyses. Subspecies delineation is based on genetic data.

| **Population ID** | **Species** | **Sample size** | **Date of sampling** | **Locality** | **Coordinates** |
| --- | --- | --- | --- | --- | --- |
| 1 | *R. riparia* | 10 | 2019/5/29 | Baisha Mountain, Buerjin,Altay, Xinjiang Uygur  Autonomous Region, PR China | 48°1'58''N, 86°51'41''E |
| 2 | *R. riparia* | 10 | 2018/6/25 | Dzharakhain Urto (Jaraakhain Urtuu), Mongolia | 49°28.4560'N, 114°05.9451'E |
| 3 | *R. riparia* | 10 | 2017/5/20 | Xingzheng District, Zhengzhou, Henan Province, PR China | 34°23'N, 113°48'E |
| 4 | *R. d. diluta* | 9 | 2019/5/10 | Alar, Xinjiang Uygur Autonomous Region, PR China | 40°35.433'N, 81°43.39'E |
| 5 | *R. d. diluta* | 10 | 2019/5/12 | Khotan, Xinjiang Uygur Autonomous Region, PR China | 37°11'39.51"N, 79°57'41.05''E |
| 6 | *R. d. diluta* | 8 | 2017/5/27 | Wujiaqu, Changji, Xinjiang Uygur Autonomous Region, PR China | 44°22'N, 87°53'E |
| 7 | *R. d. diluta* | 10 | 2019/5/22 | Yining, Xinjiang Uygur Autonomous Region, PR China | 43°54'43.92"N, 81°14'8.52"E |
| 8a | *R. d. tibetana* | 18 | 2018/6/21 | Elsen Tasarkhai, Mongolia | 47°21.5352'N, 103°41.9692'E |
| 8b | *R. d. tibetana* | 6 | 2018/6/18 | Argalant, Mongolia | 47°52.4902'N, 105°47.7577'E |
| 9a | *R. d. tibetana* | 5 | 2018/7/5 | Zoige, Sichuan Province, PR China | 33°27'12”N, 102°28'55”E |
| 9b | *R. d. tibetana* | 5 | 2018/6/22 | Gahai Lake, Gansu Province, PR China | 34°14'50‘’N, 102°20'32‘’E |
| 10a | *R. d. tibetana* | 5 | 2018/6/29 | Qinghai Lake, Qinghai Province, PR China | 36°34'15‘’N, 100°44'39‘’E |
| 10b | *R. d. tibetana* | 4 | 2018/6/27 | Rubber Mountain, Qinghai Province, PR China | 36°45'55''N, 99°38'20''E |
| 11 | *R. d. tibetana* | 10 | 2018/6/6 | Qushui County, Lhasa, Tibet autonomous region, PR China | 29°20'13"N, 90°52'36''E |
| 12 | *R. d. fohkienensis* | 10 | 2017/5/21 | Meixian county, Shaanxi Province, PR China | 34°14'N, 107°54'E |
| 13 | *R. d. fohkienensis* | 10 | 2016/4/26 | Yongjia Academy, Yongjia County, Wenzhou, Zhejiang Province, PR China | 28°16'9.66"N, 120°40'54.89"E |
| 14 | *R. d. fohkienensis* | 9 | 2018/4/29 | Lanxi County, Yibin, Sichuan, PR China | 28°48'45.90"N, 104°57'1.65"E |

Table S2 Specimens sampled, museum number or sample name, collection locality, GenBank accession numbers for the of ND2 haplotype sequences and accessions for NCBI sequence read archive (SRA) under BioProject PRJNA755835 of each individual.

| **Species** | **Museum number** | **Locality** | **ND2** | **SRA** |
| --- | --- | --- | --- | --- |
| *R. d. diluta* | SYSb006513 | Alar, Xinjiang, PR China | MZ747658 | SAMN20856160 |
| *R. d. diluta* | SYSb006515 | Alar, Xinjiang, PR China | MZ747659 | SAMN20856161 |
| *R. d. diluta* | SYSb006517 | Alar, Xinjiang, PR China | MZ747657 | SAMN20856162 |
| *R. d. diluta* | SYSb006518 | Alar, Xinjiang, PR China | MZ747657 | SAMN20856163 |
| *R. d. diluta* | SYSb006521 | Alar, Xinjiang, PR China | MZ747656 | SAMN20856164 |
| *R. d. diluta* | SYSb006522 | Alar, Xinjiang, PR China | MZ747657 | SAMN20856165 |
| *R. d. diluta* | SYSb006755 | Alar, Xinjiang, PR China | MZ747658 | SAMN20856166 |
| *R. d. diluta* | SYSb006756 | Alar, Xinjiang, PR China | MZ747657 | SAMN20856167 |
| *R. d. diluta* | SYSb007331 | Alar, Xinjiang, PR China | MZ747657 | SAMN20856168 |
| *R. d. diluta* | SYSb007336 | Khotan, Xinjiang, PR China | MZ747657 | SAMN20856169 |
| *R. d. diluta* | SYSb007338 | Khotan, Xinjiang, PR China | MZ747660 | SAMN20856170 |
| *R. d. diluta* | SYSb007342 | Khotan, Xinjiang, PR China | MZ747657 | SAMN20856171 |
| *R. d. diluta* | SYSb007343 | Khotan, Xinjiang, PR China | MZ747657 | SAMN20856172 |
| *R. d. diluta* | SYSb007345 | Khotan, Xinjiang, PR China | MZ747657 | SAMN20856173 |
| *R. d. diluta* | SYSb007348 | Khotan, Xinjiang, PR China | MZ747657 | SAMN20856174 |
| *R. d. diluta* | SYSb007349 | Khotan, Xinjiang, PR China | MZ747657 | SAMN20856175 |
| *R. d. diluta* | SYSb007351 | Khotan, Xinjiang, PR China | MZ747657 | SAMN20856176 |
| *R. d. diluta* | SYSb007352 | Khotan, Xinjiang, PR China | MZ747657 | SAMN20856177 |
| *R. d. diluta* | SYSb007962 | Khotan, Xinjiang, PR China | MZ747661 | SAMN20856178 |
| *R. d. diluta* | SYSb007964 | Yining, Xinjiang, PR China | MZ747664 | SAMN20856179 |
| *R. d. diluta* | SYSb007965 | Yining, Xinjiang, PR China | MZ747665 | SAMN20856180 |
| *R. d. diluta* | SYSb007967 | Yining, Xinjiang, PR China | MZ747662 | SAMN20856181 |
| *R. d. diluta* | SYSb008009 | Yining, Xinjiang, PR China | MZ747662 | SAMN20856182 |
| *R. d. diluta* | SYSb008334 | Yining, Xinjiang, PR China | MZ747657 | SAMN20856183 |
| *R. d. diluta* | SYSb008336 | Yining, Xinjiang, PR China | MZ747657 | SAMN20856184 |
| *R. d. diluta* | SYSb008339 | Yining, Xinjiang, PR China | MZ747657 | SAMN20856185 |
| *R. d. diluta* | SYSb008340 | Yining, Xinjiang, PR China | MZ747657 | SAMN20856186 |
| *R. d. diluta* | SYSb008343 | Yining, Xinjiang, PR China | MZ747657 | SAMN20856187 |
| *R. d. diluta* | SYSb008344 | Yining, Xinjiang, PR China | MZ747660 | SAMN20856188 |
| *R. d. fohkienensis* | SYSb003791 | Yongjia, Wenzhou, Zhejiang, PR China | MZ747666 | SAMN20856189 |
| *R. d. fohkienensis* | SYSb003792 | Yongjia, Wenzhou, Zhejiang, PR China | MZ747666 | SAMN20856190 |
| *R. d. fohkienensis* | SYSb003793 | Yongjia, Wenzhou, Zhejiang, PR China | MZ747666 | SAMN20856191 |
| *R. d. fohkienensis* | SYSb003794 | Yongjia, Wenzhou, Zhejiang, PR China | MZ747666 | SAMN20856192 |
| *R. d. fohkienensis* | SYSb003795 | Yongjia, Wenzhou, Zhejiang, PR China | MZ747668 | SAMN20856193 |
| *R. d. fohkienensis* | SYSb003796 | Yongjia, Wenzhou, Zhejiang, PR China | MZ747666 | SAMN20856194 |
| *R. d. fohkienensis* | SYSb003797 | Yongjia, Wenzhou, Zhejiang, PR China | MZ747666 | SAMN20856195 |
| *R. d. fohkienensis* | SYSb003798 | Yongjia, Wenzhou, Zhejiang, PR China | MZ747669 | SAMN20856196 |
| *R. d. fohkienensis* | SYSb003799 | Yongjia, Wenzhou, Zhejiang, PR China | MZ747666 | SAMN20856197 |
| *R. d. fohkienensis* | SYSb003800 | Yongjia, Wenzhou, Zhejiang, PR China | MZ747666 | SAMN20856198 |
| *R. d. fohkienensis* | SYSb006526 | Meixian, Shaanxi, PR China | MZ747666 | SAMN20856199 |
| *R. d. fohkienensis* | SYSb006528 | Meixian, Shaanxi, PR China | MZ747666 | SAMN20856200 |
| *R. d. fohkienensis* | SYSb006529 | Meixian, Shaanxi, PR China | MZ747666 | SAMN20856201 |
| *R. d. fohkienensis* | SYSb006531 | Meixian, Shaanxi, PR China | MZ747667 | SAMN20856202 |
| *R. d. fohkienensis* | SYSb006532 | Meixian, Shaanxi, PR China | MZ747666 | SAMN20856203 |
| *R. d. fohkienensis* | SYSb006533 | Meixian, Shaanxi, PR China | MZ747666 | SAMN20856204 |
| *R. d. fohkienensis* | SYSb006534 | Meixian, Shaanxi, PR China | MZ747666 | SAMN20856205 |
| *R. d. fohkienensis* | SYSb006535 | Meixian, Shaanxi, PR China | MZ747667 | SAMN20856206 |
| *R. d. fohkienensis* | SYSb006536 | Meixian, Shaanxi, PR China | MZ747666 | SAMN20856207 |
| *R. d. fohkienensis* | SYSb006537 | Meixian, Shaanxi, PR China | MZ747666 | SAMN20856208 |
| *R. d. diluta* | SYSb006540 | Wujiaqu, Xinjiang, PR China | MZ747662 | SAMN20856209 |
| *R. d. diluta* | SYSb006541 | Wujiaqu, Xinjiang, PR China | MZ747663 | SAMN20856210 |
| *R. d. diluta* | SYSb006542 | Wujiaqu, Xinjiang, PR China | MZ747663 | SAMN20856211 |
| *R. d. diluta* | SYSb006543 | Wujiaqu, Xinjiang, PR China | MZ747657 | SAMN20856212 |
| *R. d. diluta* | SYSb006544 | Wujiaqu, Xinjiang, PR China | MZ747661 | SAMN20856213 |
| *R. d. diluta* | SYSb006545 | Wujiaqu, Xinjiang, PR China | MZ747657 | SAMN20856214 |
| *R. d. diluta* | SYSb006546 | Wujiaqu, Xinjiang, PR China | MZ747657 | SAMN20856215 |
| *R. d. diluta* | SYSb006547 | Wujiaqu, Xinjiang, PR China | MZ747657 | SAMN20856216 |
| *R. d. fohkienensis* | SYSb008373 | Lanxi, Yibin, Sichuan, PR China | MZ747671 | SAMN20856217 |
| *R. d. fohkienensis* | SYSb008375 | Lanxi, Yibin, Sichuan, PR China | MZ747666 | SAMN20856218 |
| *R. d. fohkienensis* | SYSb008380 | Lanxi, Yibin, Sichuan, PR China | MZ747666 | SAMN20856219 |
| *R. d. fohkienensis* | SYSb008381 | Lanxi, Yibin, Sichuan, PR China | MZ747666 | SAMN20856220 |
| *R. d. fohkienensis* | SYSb008382 | Lanxi, Yibin, Sichuan, PR China | MZ747666 | SAMN20856221 |
| *R. d. fohkienensis* | SYSb008384 | Lanxi, Yibin, Sichuan, PR China | MZ747666 | SAMN20856222 |
| *R. d. fohkienensis* | SYSb008385 | Lanxi, Yibin, Sichuan, PR China | MZ747666 | SAMN20856223 |
| *R. d. fohkienensis* | SYSb008388 | Lanxi, Yibin, Sichuan, PR China | MZ747666 | SAMN20856224 |
| *R. d. fohkienensis* | SYSb008391 | Lanxi, Yibin, Sichuan, PR China | MZ747670 | SAMN20856225 |
| *R. d. tibetana* | SYSb003782 | East Huanhu Road, Qinghai, PR China | MZ747672 | SAMN20856226 |
| *R. d. tibetana* | SYSb003783 | East Huanhu Road, Qinghai, PR China | MZ747673 | SAMN20856227 |
| *R. d. tibetana* | SYSb003941 | Lhasa, Tibet, PR China | MZ747674 | SAMN20856228 |
| *R. d. tibetana* | SYSb003942 | Lhasa, Tibet, PR China | MZ747679 | SAMN20856229 |
| *R. d. tibetana* | SYSb003943 | Lhasa, Tibet, PR China | MZ747674 | SAMN20856230 |
| *R. d. tibetana* | SYSb003945 | Lhasa, Tibet, PR China | MZ747672 | SAMN20856231 |
| *R. d. tibetana* | SYSb003968 | Lhasa, Tibet, PR China | MZ747672 | SAMN20856232 |
| *R. d. tibetana* | SYSb004145 | Lhasa, Tibet, PR China | MZ747677 | SAMN20856233 |
| *R. d. tibetana* | SYSb004151 | Lhasa, Tibet, PR China | MZ747674 | SAMN20856234 |
| *R. d. tibetana* | SYSb004153 | Lhasa, Tibet, PR China | MZ747678 | SAMN20856235 |
| *R. d. tibetana* | SYSb004154 | Lhasa, Tibet, PR China | MZ747672 | SAMN20856236 |
| *R. d. tibetana* | SYSb004156 | Lhasa, Tibet, PR China | MZ747672 | SAMN20856237 |
| *R. d. tibetana* | SYSb004163 | Gahai Lake, Gansu, PR China | MZ747675 | SAMN20856238 |
| *R. d. tibetana* | SYSb004164 | Gahai Lake, Gansu, PR China | MZ747672 | SAMN20856239 |
| *R. d. tibetana* | SYSb004169 | Gahai Lake, Gansu, PR China | MZ747672 | SAMN20856240 |
| *R. d. tibetana* | SYSb004187 | Gahai Lake, Gansu, PR China | MZ747676 | SAMN20856241 |
| *R. d. tibetana* | SYSb004192 | Gahai Lake, Gansu, PR China | MZ747672 | SAMN20856242 |
| *R. d. tibetana* | SYSb004194 | Rubber Mountain, Qinghai, PR China | MZ747674 | SAMN20856243 |
| *R. d. tibetana* | SYSb004197 | Rubber Mountain, Qinghai, PR China | MZ747674 | SAMN20856244 |
| *R. d. tibetana* | SYSb004198 | Rubber Mountain, Qinghai, PR China | MZ747672 | SAMN20856245 |
| *R. d. tibetana* | SYSb004200 | Rubber Mountain, Qinghai, PR China | MZ747672 | SAMN20856246 |
| *R. d. tibetana* | SYSb005922 | East Huanhu Road, Qinghai, PR China | MZ747674 | SAMN20856247 |
| *R. d. tibetana* | SYSb005923 | East Huanhu Road, Qinghai, PR China | MZ747672 | SAMN20856248 |
| *R. d. tibetana* | SYSb005924 | East Huanhu Road, Qinghai, PR China | MZ747672 | SAMN20856249 |
| *R. d. tibetana* | SYSb005934 | Zoige, Sichuan, PR China | MZ747672 | SAMN20856250 |
| *R. d. tibetana* | SYSb005939 | Zoige, Sichuan, PR China | MZ747680 | SAMN20856251 |
| *R. d. tibetana* | SYSb003656 | Zoige, Sichuan PR China | MZ747674 | SAMN20856252 |
| *R. d. tibetana* | SYSb006084 | Zoige, Sichuan, PR China | MZ747672 | SAMN20856253 |
| *R. d. tibetana* | SYSb006087 | Zoige, Sichuan, PR China | MZ747672 | SAMN20856254 |
| *R. d. tibetana* | NMBE1078288 | Elsen Tasarkhai, Mongolia | MZ747681 | SAMN20856255 |
| *R. d. tibetana* | NMBE1078289 | Argalant, Mongolia | MZ747697 | SAMN20856256 |
| *R. d. tibetana* | NMBE1078290 | Argalant, Mongolia | MZ747674 | SAMN20856257 |
| *R. d. tibetana* | NMBE1078291 | Argalant, Mongolia | MZ747681 | SAMN20856258 |
| *R. d. tibetana* | NMBE1078292 | Argalant, Mongolia | MZ747683 | SAMN20856259 |
| *R. d. tibetana* | NMBE1078293 | Argalant, Mongolia | MZ747674 | SAMN20856260 |
| *R. d. tibetana* | NMBE1078294 | Argalant, Mongolia | MZ747681 | SAMN20856261 |
| *R. d. tibetana* | NMBE1078295 | Elsen Tasarkhai, Mongolia | MZ747674 | SAMN20856262 |
| *R. d. tibetana* | NMBE1078296 | Elsen Tasarkhai, Mongolia | MZ747685 | SAMN20856263 |
| *R. d. tibetana* | NMBE1078297 | Elsen Tasarkhai, Mongolia | MZ747682 | SAMN20856264 |
| *R. d. tibetana* | NMBE1078298 | Elsen Tasarkhai, Mongolia | MZ747674 | SAMN20856265 |
| *R. d. tibetana* | NMBE1078299 | Elsen Tasarkhai, Mongolia | MZ747683 | SAMN20856266 |
| *R. d. tibetana* | NMBE1078300 | Elsen Tasarkhai, Mongolia | MZ747674 | SAMN20856267 |
| *R. d. tibetana* | NMBE1078301 | Elsen Tasarkhai, Mongolia | MZ747681 | SAMN20856268 |
| *R. d. tibetana* | NMBE1078302 | Elsen Tasarkhai, Mongolia | MZ747683 | SAMN20856269 |
| *R. d. tibetana* | NMBE1078303 | Elsen Tasarkhai, Mongolia | MZ747683 | SAMN20856270 |
| *R. d. tibetana* | NMBE1078304 | Elsen Tasarkhai, Mongolia | MZ747683 | SAMN20856271 |
| *R. d. tibetana* | NMBE1078305 | Elsen Tasarkhai, Mongolia | MZ747684 | SAMN20856272 |
| *R. d. tibetana* | NMBE1078306 | Elsen Tasarkhai, Mongolia | MZ747683 | SAMN20856273 |
| *R. d. tibetana* | NMBE1078307 | Elsen Tasarkhai, Mongolia | MZ747674 | SAMN20856274 |
| *R. d. tibetana* | NMBE1078287 | Elsen Tasarkhai, Mongolia | MZ747683 | SAMN20856275 |
| *R. d. tibetana* | NMBE1078308 | Elsen Tasarkhai, Mongolia | MZ747674 | SAMN20856276 |
| *R. d. tibetana* | NMBE1078309 | Elsen Tasarkhai, Mongolia | MZ747681 | SAMN20856277 |
| *R. d. tibetana* | NMBE1078310 | Elsen Tasarkhai, Mongolia | MZ747674 | SAMN20856278 |
| *R. riparia* | SYSb008345 | Baisha Mountain, Altay, Xiniiang, PR China | MZ747686 | SAMN20856279 |
| *R. riparia* | SYSb008346 | Baisha Mountain, Altay, Xiniiang, PR China | MZ747687 | SAMN20856280 |
| *R. riparia* | SYSb008349 | Baisha Mountain, Altay, Xiniiang, PR China | MZ747688 | SAMN20856281 |
| *R. riparia* | SYSb008351 | Baisha Mountain, Altay, Xiniiang, PR China | MZ747688 | SAMN20856282 |
| *R. riparia* | SYSb008352 | Baisha Mountain, Altay, Xiniiang, PR China | MZ747688 | SAMN20856283 |
| *R. riparia* | SYSb008353 | Baisha Mountain, Altay, Xiniiang, PR China | MZ747689 | SAMN20856284 |
| *R. riparia* | SYSb008355 | Baisha Mountain, Altay, Xiniiang, PR China | MZ747690 | SAMN20856285 |
| *R. riparia* | SYSb008356 | Baisha Mountain, Altay, Xiniiang, PR China | MZ747688 | SAMN20856286 |
| *R. riparia* | SYSb008357 | Baisha Mountain, Altay, Xiniiang, PR China | MZ747688 | SAMN20856287 |
| *R. riparia* | SYSb008358 | Baisha Mountain, Altay, Xiniiang, PR China | MZ747688 | SAMN20856288 |
| *R. riparia* | NMBE1078312 | Dzharakhain Urto (Jaraakhain Urtuu), Mongolia | MZ747688 | SAMN20856289 |
| *R. riparia* | NMBE1078313 | Dzharakhain Urto (Jaraakhain Urtuu), Mongolia | MZ747691 | SAMN20856290 |
| *R. riparia* | NMBE1078314 | Dzharakhain Urto (Jaraakhain Urtuu), Mongolia | MZ747692 | SAMN20856291 |
| *R. riparia* | NMBE1078315 | Dzharakhain Urto (Jaraakhain Urtuu), Mongolia | MZ747688 | SAMN20856292 |
| *R. riparia* | NMBE1078316 | Dzharakhain Urto (Jaraakhain Urtuu), Mongolia | MZ747693 | SAMN20856293 |
| *R. riparia* | NMBE1078318 | Dzharakhain Urto (Jaraakhain Urtuu), Mongolia | MZ747688 | SAMN20856294 |
| *R. riparia* | NMBE1078319 | Dzharakhain Urto (Jaraakhain Urtuu), Mongolia | MZ747693 | SAMN20856295 |
| *R. riparia* | NMBE1078322 | Dzharakhain Urto (Jaraakhain Urtuu), Mongolia | MZ747688 | SAMN20856296 |
| *R. riparia* | NMBE1078323 | Dzharakhain Urto (Jaraakhain Urtuu), Mongolia | MZ747693 | SAMN20856297 |
| *R. riparia* | NMBE1078327 | Dzharakhain Urto (Jaraakhain Urtuu), Mongolia | MZ747688 | SAMN20856298 |
| *R. riparia* | SYSb006501 | Zhengzhou, Henan, PR China | MZ747694 | SAMN20856299 |
| *R. riparia* | SYSb006502 | Zhengzhou, Henan, PR China | MZ747695 | SAMN20856300 |
| *R. riparia* | SYSb006503 | Zhengzhou, Henan, PR China | MZ747693 | SAMN20856301 |
| *R. riparia* | SYSb006504 | Zhengzhou, Henan, PR China | MZ747693 | SAMN20856302 |
| *R. riparia* | SYSb006505 | Zhengzhou, Henan, PR China | MZ747693 | SAMN20856303 |
| *R. riparia* | SYSb006506 | Zhengzhou, Henan, PR China | MZ747696 | SAMN20856304 |
| *R. riparia* | SYSb006507 | Zhengzhou, Henan, PR China | MZ747688 | SAMN20856305 |
| *R. riparia* | SYSb006508 | Zhengzhou, Henan, PR China | MZ747693 | SAMN20856306 |
| *R. riparia* | SYSb006509 | Zhengzhou, Henan, PR China | MZ747696 | SAMN20856307 |
| *R. riparia* | SYSb006510 | Zhengzhou, Henan, PR China | MZ747693 | SAMN20856308 |
| *R. d. indica* | NHMUK1949.W.1.5879 | Jhelum, Punjab, Pakistan | MG881167 |  |
| *Hirundo rustica* |  |  | DQ176515 |  |

Table S3 Morphological data. All the measurements are shown in mm. The measurements below are bill to feathering (BF), bill depth (BP), bill width (BW), wing length (WL), length of P8 (P8), tail length (Ltail), length of tail fork (LF) and length of tarsus (LTar).

| **Sample ID** | **Species** | **BF** | **BP** | **BW** | **WL** | **P8** | **Ltail** | **LF** | **LTar** | **Coordinates** |
| --- | --- | --- | --- | --- | --- | --- | --- | --- | --- | --- |
| SYSb003656 | *R. d. tibetana* | 5.7 | 1.9 | 3.6 | 108 | 84 | 53 | 6.8 | 11.2 | 33°27'12N, 102°28'55E |
| SYSb003657 | *R. d. tibetana* | 6 | 2.2 | 3.3 | 110 | 85 | 54 | 7.7 | 10.8 | 29°41.273'N, 91°18.513'E |
| SYSb003941 | *R. d. tibetana* | 6.8 | 2.2 | 4.3 | 106 | 89 | 47 | 2.2 | 11.6 | 29°41.273'N, 91°18.513'E |
| SYSb003942 | *R. d. tibetana* | 5.7 | 2 | 3.5 | 109 | 84 | 56 | 4 | 11 | 29°41.273'N, 91°18.513'E |
| SYSb003943 | *R. d. tibetana* | 5.3 | 2.1 | 3.4 | 107 | 83 | 51 | 5.5 | 11.3 | 29°41.273'N, 91°18.513'E |
| SYSb003944 | *R. d. tibetana* | 5.6 | 2.2 | 3.8 | 107 | 84 | 53 | 7 | 11.1 | 29°41.273'N, 91°18.513'E |
| SYSb003945 | *R. d. tibetana* | 5.9 | 2.3 | 3.7 | 110 | 80 | 53 | 5.2 | 11.6 | 29°20'12.8”N, 90°52'36.8"E |
| SYSb003968 | *R. d. tibetana* | 5.2 | 2.1 | 3.4 | 106 | 84 | 52 | 7.7 | 11.1 | 29°20'12.8”N, 90°52'36.8"E |
| SYSb004104 | *R. d. tibetana* | 6.3 | 2.2 | 3.9 | 111 | 86 | 52 | 5.4 | 11 | 29°20'12.8”N, 90°52'36.8"E |
| SYSb004145 | *R. d. tibetana* | 5.8 | 2.2 | 3.9 | 110 | 84 | 48 | 3.1 | 10 | 29°20'12.8”N, 90°52'36.8"E |
| SYSb004146 | *R. d. tibetana* | 5.8 | 2.3 | 4.5 | 109 | 83 | 49 | 1.8 | 10.5 | 29°20'12.8”N, 90°52'36.8"E |
| SYSb004147 | *R. d. tibetana* | 5.8 | 2.3 | 4.1 | 108 | 83 | 50 | 2.2 | 10.8 | 29°20'12.8”N, 90°52'36.8"E |
| SYSb004148 | *R. d. tibetana* | 4.1 | 2.4 | 3.7 | 102 | 81 | 49 | 3 | 9.8 | 29°20'12.8”N, 90°52'36.8"E |
| SYSb004149 | *R. d. tibetana* | 5.9 | 2.2 | 2.8 | 107 | 83 | 49 | 7.8 | 10.8 | 29°20'12.8”N, 90°52'36.8"E |
| SYSb004150 | *R. d. tibetana* | 5.3 | 2.3 | 4.1 | 107 | 82 | 49 | 6.1 | 11.4 | 29°20'12.8”N, 90°52'36.8"E |
| SYSb004151 | *R. d. tibetana* | 6.1 | 2.5 | 4.2 | 109 | 83 | 52 | 4.2 | 10.8 | 29°20'12.8”N, 90°52'36.8"E |
| SYSb004152 | *R. d. tibetana* | 6 | 2.3 | 4 | 104 | 82 | 52 | 7.2 | 11.8 | 29°20'12.8”N, 90°52'36.8"E |
| SYSb004153 | *R. d. tibetana* | 6.4 | 2.3 | 4.4 | 112 | 84 | 58 | 3.8 | 11.7 | 29°20'12.8”N, 90°52'36.8"E |
| SYSb004154 | *R. d. tibetana* | 5.8 | 2.1 | 4 | 107 | 81 | 49 | 2.3 | 11.8 | 29°20'12.8”N, 90°52'36.8"E |
| SYSb004155 | *R. d. tibetana* | 6.1 | 2.3 | 4.1 | 110 | 85 | 50 | 5.9 | 11.8 | 29°20'12.8”N, 90°52'36.8"E |
| SYSb004156 | *R. d. tibetana* | 6.1 | 2.3 | 4.1 | 111 | 84 | 51 | 1.8 | 11.8 | 29°20'12.8”N, 90°52'36.8"E |
| SYSb004157 | *R. d. tibetana* | 6.1 | 2.2 | 4.5 | 110 | 83.5 | 52 | 4.1 | 11.6 | 34°14'49.6"N, 102°20'31.5"E |
| SYSb004158 | *R. d. tibetana* | 6.1 | 2.3 | 3.8 | 109 | 85 | 53 | 5 | 11.9 | 34°14'49.6"N, 102°20'31.5"E |
| SYSb004159 | *R. d. tibetana* | 5.4 | 2 | 3.6 | 108 | 84 | 51 | 6.2 | 11.7 | 34°14'49.6"N, 102°20'31.5"E |
| SYSb004160 | *R. d. tibetana* | 5.8 | 2.1 | 3.8 | 116 | 87 | 53 | 5.5 | 11.7 | 34°14'49.6"N, 102°20'31.5"E |
| SYSb004161 | *R. d. tibetana* | 5.9 | 2.2 | 4.3 | 111 | 84 | 53 | 3.1 | 11.7 | 34°14'49.6"N, 102°20'31.5"E |
| SYSb004162 | *R. d. tibetana* | 6.2 | 2.3 | 4.5 | 114 | 87 | 55 | 4.9 | 11.8 | 34°14'49.6"N, 102°20'31.5"E |
| SYSb004163 | *R. d. tibetana* | 6.1 | 2.2 | 4.3 | 103 | 83 | 51 | 4.4 | 11.8 | 34°14'49.6"N, 102°20'31.5"E |
| SYSb004164 | *R. d. tibetana* | 6.1 | 2.2 | 4.1 | 111 | 83 | 51 | 4.9 | 11.8 | 34°14'49.6"N, 102°20'31.5"E |
| SYSb004165 | *R. d. tibetana* | 5.8 | 2 | 4.5 | 109 | 86 | 53 | 7.6 | 11.7 | 34°14'49.6"N, 102°20'31.5"E |
| SYSb004166 | *R. d. tibetana* | 5.7 | 2.3 | 4.2 | 111 | 83 | 53 | 5.4 | 11.2 | 34°14'49.6"N, 102°20'31.5"E |
| SYSb004167 | *R. d. tibetana* | 6.2 | 2.1 | 4 | 107 | 85 | 51 | 4.5 | 9.9 | 34°14'49.6"N, 102°20'31.5"E |
| SYSb004168 | *R. d. tibetana* | 5.2 | 2 | 4.6 | 109 | 84 | 51 | 3.7 | 11.7 | 34°14'49.6"N, 102°20'31.5"E |
| SYSb004169 | *R. d. tibetana* | 6.2 | 2.2 | 4.5 | 108 | 83 | 51 | 7.2 | 11.8 | 34°14'49.6"N, 102°20'31.5"E |
| SYSb004170 | *R. d. tibetana* | 6.3 | 2.1 | 3.7 | 112 | 84 | 51 | 3.2 | 11.8 | 34°14'49.6"N, 102°20'31.5"E |
| SYSb004187 | *R. d. tibetana* | 6.1 | 2.2 | 3.7 | 113 | 88 | 55 | 6 | 11.7 | 34°14'49.6"N, 102°20'31.5"E |
| SYSb004188 | *R. d. tibetana* | 5.4 | 2.2 | 4 | 112 | 85 | 50 | 3.9 | 11.7 | 34°14'49.6"N, 102°20'31.5"E |
| SYSb004189 | *R. d. tibetana* | 5.1 | 2.5 | 4.1 | 114 | 87 | 52 | 2 | 11.7 | 34°14'49.6"N, 102°20'31.5"E |
| SYSb004190 | *R. d. tibetana* | 6.1 | 2.2 | 4 | 111 | 83 | 50 | 5.2 | 11.7 | 34°14'49.6"N, 102°20'31.5"E |
| SYSb004191 | *R. d. tibetana* | 6.1 | 2 | 3.4 | 109 | 86 | 52 | 4.2 | 11.8 | 34°14'49.6"N, 102°20'31.5"E |
| SYSb004192 | *R. d. tibetana* | 6.2 | 2.2 | 3.2 | 110 | 85 | 50 | 5.8 | 11.8 | 34°14'49.6"N, 102°20'31.5"E |
| SYSb004193 | *R. d. tibetana* | 5.9 | 2.2 | 3.5 | 113 | 87 | 52 | 3 | 11.8 | 34°14'49.6"N, 102°20'31.5"E |
| SYSb004194 | *R. d. tibetana* | 6.5 | 2.2 | 4 | 107 | 82 | 51 | 5.3 | 11.7 | 36°45'54.9"N, 99°38'20.1"E |
| SYSb004195 | *R. d. tibetana* | 5.2 | 2.3 | 3.7 | 107 | 82 | 52 | 6.1 | 10.9 | 36°45'54.9"N, 99°38'20.1"E |
| SYSb004196 | *R. d. tibetana* | 5.5 | 2.1 | 3.3 | 114 | 85 | 53 | 6.7 | 10 | 36°45'54.9"N, 99°38'20.1"E |
| SYSb004197 | *R. d. tibetana* | 6.1 | 2.1 | 3 | 114 | 88 | 55 | 6.8 | 11.7 | 36°45'54.9"N, 99°38'20.1"E |
| SYSb004198 | *R. d. tibetana* | 6.1 | 2.1 | 3.1 | 108 | 84 | 52 | 4.2 | 11.8 | 36°45'54.9"N, 99°38'20.1"E |
| SYSb004199 | *R. d. tibetana* | 5.8 | 2.3 | 3.8 | 112 | 85.5 | 55 | 6.4 | 11.9 | 36°45'54.9"N, 99°38'20.1"E |
| SYSb004200 | *R. d. tibetana* | 6.7 | 2.3 | 4 | 108 | 85 | 54 | 8.4 | 11.7 | 36°45'54.9"N, 99°38'20.1"E |
| SYSb004365 | *R. d. tibetana* | 6.1 | 2.2 | 3.7 | 111 | 86.5 | 52 | 5.2 | 10.9 | 36°45'54.9"N, 99°38'20.1"E |
| SYSb005920 | *R. d. tibetana* | 6.2 | 2.1 | 3.4 | 111 | 86 | 52 | 3.1 | 11.3 | 36°45'54.9"N, 99°38'20.1"E |
| SYSb005921 | *R. d. tibetana* | 6.1 | 2.2 | 3.7 | 113.5 | 88 | 52 | 2.8 | 11.6 | 36°45'54.9"N, 99°38'20.1"E |
| SYSb005922 | *R. d. tibetana* | 6.2 | 2.1 | 3.7 | 107 | 82 | 50 | 6.6 | 9.8 | 36° 34'15.3"N, 100°44'38.7"E |
| SYSb005923 | *R. d. tibetana* | 5.9 | 2.3 | 3.7 | 111 | 84 | 49 | 5.5 | 11.7 | 36° 34'15.3"N, 100°44'38.7"E |
| SYSb005925 | *R. d. tibetana* | 6.1 | 2.2 | 3.1 | 106 | 82 | 50 | 5.5 | 11.2 | 36°50'10.1"N, 99°43'14.2"E |
| SYSb005926 | *R. d. tibetana* | 6 | 2.3 | 4 | 113 | 87 | 54 | 6.1 | 11.7 | 36°50'10.1"N, 99°43'14.2"E |
| SYSb005927 | *R. d. tibetana* | 6 | 2.3 | 3.3 | 116 | 83 | 52 | 5.5 | 11 | 36°50'10.1"N, 99°43'14.2"E |
| SYSb005928 | *R. d. tibetana* | 6 | 2.2 | 3.5 | 110 | 84.5 | 51 | 4.9 | 11.2 | 36°50'10.1"N, 99°43'14.2"E |
| SYSb005929 | *R. d. tibetana* | 6 | 2.2 | 4.1 | 109.5 | 83.5 | 52 | 2.5 | 11.7 | 36°50'10.1"N, 99°43'14.2"E |
| SYSb005930 | *R. d. tibetana* | 6.2 | 2.2 | 4.3 | 111 | 85 | 52 | 5.3 | 11.8 | 33°34'1.1"N, 102°28'35.4"E |
| SYSb005931 | *R. d. tibetana* | 5.9 | 2 | 4.8 | 112 | 83.5 | 50 | 2.3 | 11.8 | 33°34'1.1"N, 102°28'35.4"E |
| SYSb005932 | *R. d. tibetana* | 5.7 | 2.3 | 3.5 | 113 | 85 | 54 | 3 | 11.2 | 33°34'1.1"N, 102°28'35.4"E |
| SYSb005933 | *R. d. tibetana* | 6.3 | 2.5 | 3.8 | 109 | 83 | 52 | 4.5 | 11.8 | 33°34'1.1"N, 102°28'35.4"E |
| SYSb005934 | *R. d. tibetana* | 5.9 | 2.4 | 3.8 | 106 | 78 | 50 | 4.7 | 11.2 | 33°34'1.1"N, 102°28'35.4"E |
| SYSb005935 | *R. d. tibetana* | 5.9 | 2.1 | 4.1 | 112 | 85 | 53 | 6.5 | 11.3 | 33°34'1.1"N, 102°28'35.4"E |
| SYSb005936 | *R. d. tibetana* | 6.7 | 2.2 | 3.7 | 112 | 86 | 52 | 6.4 | 11.7 | 33°34'1.1"N, 102°28'35.4"E |
| SYSb005937 | *R. d. tibetana* | 6 | 2.2 | 3.8 | 111 | 80 | 54 | 5.7 | 11.7 | 33°34'1.1"N, 102°28'35.4"E |
| SYSb005938 | *R. d. tibetana* | 6.1 | 2.2 | 3.2 | 119 | 82 | 51 | 5.6 | 11.8 | 33°34'1.1"N, 102°28'35.4"E |
| SYSb005939 | *R. d. tibetana* | 6.4 | 2.3 | 3.4 | 114 | 86 | 55 | 5.3 | 11.5 | 33°34'1.1"N, 102°28'35.4"E |
| SYSb006050 | *R. d. tibetana* | 6.8 | 2.4 | 3.9 | 108 | 88 | 51 | 2.8 | 11.7 | 33°34'1.1"N, 102°28'35.4"E |
| SYSb006051 | *R. d. tibetana* | 6.1 | 2 | 3.5 | 116 | 90 | 56 | 4.4 | 11.7 | 33°34'1.1"N, 102°28'35.4"E |
| SYSb006053 | *R. d. tibetana* | 6.2 | 2.1 | 4.2 | 109 | 82 | 51 | 4.1 | 11.7 | 33°34'1.1"N, 102°28'35.4"E |
| SYSb006084 | *R. d. tibetana* | 6.2 | 2.4 | 3.8 | 114 | 83 | 52 | 5.1 | 11.4 | 33°34'1.1"N, 102°28'35.4"E |
| SYSb006085 | *R. d. tibetana* | 6.4 | 2.2 | 3.6 | 118 | 83 | 49 | 4.2 | 11.7 | 33°34'1.1"N, 102°28'35.4"E |
| SYSb006086 | *R. d. tibetana* | 6.2 | 2.1 | 3.8 | 110 | 83 | 50 | 5.2 | 11.7 | 33°34'1.1"N, 102°28'35.4"E |
| SYSb006087 | *R. d. tibetana* | 6.7 | 2.3 | 4 | 111 | 85 | 52 | 5.6 | 11.7 | 33°34'1.1"N, 102°28'35.4"E |
| SYSb006088 | *R. d. tibetana* | 6.3 | 2 | 4.1 | 110 | 85 | 55 | 5.9 | 11.6 | 33°34'1.1"N, 102°28'35.4"E |
| SYSb006089 | *R. d. diluta* | 4.89 | 1.8 | 3.39 | 107 | 80.5 | 51 | 5.9 | 11.78 | 44°21'49.85' N, 87°53'11.77''E |
| SYSb006090 | *R. d. diluta* | 6.7 | 2.2 | 3.7 | 106 | 80.05 | 47 | 6.08 | 10.61 | 44°21'49.85' N, 87°53'11.77''E |
| SYSb006091 | *R. d. diluta* | 6.8 | 2.2 | 3.9 | 105.5 | 81 | 51 | 8.8 | 10.1 | 44°21'49.85' N, 87°53'11.77''E |
| SYSb006092 | *R. d. diluta* | 5.7 | 1.9 | 3.9 | 102 | 79 | 53 | 7.7 | 10.65 | 44°21'49.85' N, 87°53'11.77''E |
| SYSb006093 | *R. d. diluta* | 5.9 | 2.5 | 3.59 | 109.5 | 82 | 52.5 | 7.1 | 10.9 | 44°21'49.85' N, 87°53'11.77''E |
| SYSb006094 | *R. d. diluta* | 5.8 | 2.02 | 3.73 | 105 | 80.5 | 52.5 | 5.9 | 10.3 | 44°21'49.85' N, 87°53'11.77''E |
| SYSb006095 | *R. d. diluta* | 5.7 | 2 | 3.95 | 109.5 | 83 | 52 | 7.8 | 11.35 | 44°21'49.85' N, 87°53'11.77''E |
| SYSb006096 | *R. d. diluta* | 6.15 | 1.75 | 3.6 | 106 | 80.5 | 51.5 | 5.75 | 10.5 | 44°21'49.85' N, 87°53'11.77''E |
| SYSb006098 | *R. d. diluta* | 6 | 1.9 | 3.9 | 104.5 | 79.5 | 51.5 | 6.8 | 10.7 | 44°21'49.85' N, 87°53'11.77''E |
| SYSb006099 | *R. d. diluta* | 5.1 | 1.9 | 3 | 105 | 78.5 | 49.5 | 6.3 | 8.8 | 44°21'49.85' N, 87°53'11.77''E |
| SYSb006100 | *R. d. diluta* | 5.95 | 2 | 3.75 | 105 | 81 | 51.5 | 7 | 92.5 | 44°21'49.85' N, 87°53'11.77''E |
| SYSb006514 | *R. d. diluta* | 5.7 | 1.9 | 3.45 | 108 | 82.5 | 52.5 | 7.15 | 10.7 | 40°35.433' N, 81°43.39'E |
| SYSb006515 | *R. d. diluta* | 5.2 | 1.85 | 3.1 | 105.5 | 82 | 52 | 8.85 | 10.6 | 40°35.433' N, 81°43.39'E |
| SYSb006516 | *R. d. diluta* | 5.8 | 2.1 | 3.5 | 104 | 79.5 | 51.5 | 7.95 | 10.4 | 40°35.433' N, 81°43.39'E |
| SYSb006517 | *R. d. diluta* | 6.1 | 2.25 | 4.05 | 109.5 | 83.5 | 54 | 9.15 | 10.7 | 40°35.433' N, 81°43.39'E |
| SYSb006518 | *R. d. diluta* | 5.8 | 2 | 3.6 | 105 | 80 | 49.5 | 3.8 | 10.3 | 40°35.433' N, 81°43.39'E |
| SYSb006520 | *R. d. diluta* | 5.9 | 2.05 | 3.25 | 105 | 80 | 47.5 | 6.75 | 10 | 40°35.433' N, 81°43.39'E |
| SYSb006521 | *R. d. diluta* | 6 | 1.95 | 4.45 | 104 | 80 | 42 | 5.85 | 9.65 | 40°35.433' N, 81°43.39'E |
| SYSb006522 | *R. d. diluta* | 6 | 1.9 | 3.45 | 100.5 | 76.5 | 48 | 7.8 | 9.7 | 40°35.433' N, 81°43.39'E |
| SYSb006523 | *R. d. diluta* | 5.7 | 1.6 | 3.3 | 104 | 78 | 45.5 | 6.75 | 10 | 40°35.433' N, 81°43.39'E |
| SYSb006755 | *R. d. diluta* | 5.3 | 1.95 | 4 | 107 | 82.5 | 51 | 9.6 | 9.8 | 40°35.433' N, 81°43.39'E |
| SYSb006756 | *R. d. diluta* | 5.7 | 1.9 | 3.5 | 103 | 73.5 | 49 | 7.75 | 9.7 | 40°35.433' N, 81°43.39'E |
| SYSb006757 | *R. d. diluta* | 6 | 1.75 | 3.5 | 102.5 | 79.5 | 47 | 6.7 | 10.7 | 40°35.433' N, 81°43.39'E |
| SYSb006758 | *R. d. diluta* | 5.7 | 1.9 | 3.85 | 104 | 79 | 49 | 6.15 | 10.15 | 40°35.433' N, 81°43.39'E |
| SYSb006759 | *R. d. diluta* | 5.2 | 1.75 | 3.65 | 105.5 | 81 | 44.5 | 5.2 | 10.1 | 40°35.433' N, 81°43.39'E |
| SYSb007331 | *R. d. diluta* | 6 | 1.9 | 2.95 | 101 | 78 | 47.5 | 6.3 | 10.4 | 40°35.433' N, 81°43.39'E |
| SYSb007332 | *R. d. diluta* | 5.4 | 1.8 | 3.75 | 104.5 | 77.5 | 50 | 7.7 | 10.7 | 40°35.433' N, 81°43.39'E |
| SYSb007333 | *R. d. diluta* | 5.7 | 1.75 | 3.4 | 100 | 79.5 | 49 | 5.1 | 10.85 | 40°35.433' N, 81°43.39'E |
| SYSb007334 | *R. d. diluta* | 5.8 | 1.6 | 3.2 | 106 | 81 | 49 | 5.8 | 9.5 | 40°35.433' N, 81°43.39'E |
| SYSb007335 | *R. d. diluta* | 5.6 | 1.8 | 3.8 | 105 | 81.5 | 46 | 7.7 | 10.5 | 40°35.433' N, 81°43.39'E |
| SYSb007336 | *R. d. diluta* | 5.7 | 2 | 3.4 | 103 | 78.5 | 48 | 5.8 | 10.35 | 37°11'39.51" N, 79°57'41.05''E |
| SYSb007337 | *R. d. diluta* | 6.2 | 1.9 | 3.2 | 105 | 83 | 52 | 8.2 | 9.9 | 37°11'39.51" N, 79°57'41.05''E |
| SYSb007338 | *R. d. diluta* | 5.2 | 2.1 | 4 | 99.5 | 75.5 | 45 | 5.8 | 10.7 | 37°11'39.51" N, 79°57'41.05''E |
| SYSb007340 | *R. d. diluta* | 6 | 1.9 | 4.15 | 107 | 81 | 52 | 7.7 | 10.8 | 37°11'39.51" N, 79°57'41.05''E |
| SYSb007341 | *R. d. diluta* | 5.4 | 2 | 3.35 | 106.5 | 82 | 48 | 6 | 10.3 | 37°11'39.51" N, 79°57'41.05''E |
| SYSb007342 | *R. d. diluta* | 5.1 | 2.1 | 3.4 | 104 | 79 | 48 | 6.1 | 10.8 | 37°11'39.51" N, 79°57'41.05''E |
| SYSb007343 | *R. d. diluta* | 6.2 | 2 | 4 | 103.5 | 77.5 | 47 | 7.9 | 11.1 | 37°11'39.51" N, 79°57'41.05''E |
| SYSb007344 | *R. d. diluta* | 6.9 | 2.3 | 3.8 | 109 | 82.5 | 51 | 8.7 | 10.2 | 37°11'39.51" N, 79°57'41.05''E |
| SYSb007345 | *R. d. diluta* | 6.4 | 2.1 | 3.7 | 107 | 82 | 53 | 6.1 | 10 | 37°11'39.51" N, 79°57'41.05''E |
| SYSb007346 | *R. d. diluta* | 6.2 | 2.1 | 4.2 | 105.5 | 79 | 49 | 6.9 | 10.7 | 37°11'39.51" N, 79°57'41.05''E |
| SYSb007347 | *R. d. diluta* | 6.3 | 2.1 | 3.7 | 104 | 78 | 50 | 7.7 | 10.2 | 37°11'39.51" N, 79°57'41.05''E |
| SYSb007348 | *R. d. diluta* | 5.9 | 1.9 | 3.9 | 101 | 78 | 45 | 4 | 10 | 37°11'39.51" N, 79°57'41.05''E |
| SYSb007349 | *R. d. diluta* | 6.2 | 1.95 | 3.9 | 102 | 78 | 49 | 7.8 | 10.2 | 37°11'39.51" N, 79°57'41.05''E |
| SYSb007350 | *R. d. diluta* | 5.7 | 2 | 3.4 | 104 | 80.5 | 49 | 5.9 | 10.3 | 37°11'39.51" N, 79°57'41.05''E |
| SYSb007351 | *R. d. diluta* | 6.8 | 1.9 | 4.1 | 105 | 81.5 | 53.5 | 8.5 | 10.3 | 37°11'39.51" N, 79°57'41.05''E |
| SYSb007352 | *R. d. diluta* | 5.6 | 2 | 3.5 | 102.5 | 77.8 | 48 | 7.8 | 9.9 | 37°11'39.51" N, 79°57'41.05''E |
| SYSb007353 | *R. d. diluta* | 6.7 | 2 | 3.8 | 104 | 79 | 47 | 6.8 | 10.2 | 37°11'39.51" N, 79°57'41.05''E |
| SYSb007354 | *R. d. diluta* | 5.7 | 1.9 | 3.8 | 104 | 81 | 51 | 6.7 | 10.8 | 37°11'39.51" N, 79°57'41.05''E |
| SYSb007962 | *R. d. diluta* | 6.1 | 2 | 4.2 | 106 | 79.5 | 53 | 6.9 | 10.2 | 37°11'39.51" N, 79°57'41.05''E |
| SYSb007963 | *R. d. diluta* | 6.2 | 2 | 4.15 | 104 | 78 | 47 | 6.7 | 10.2 | 37°11'39.51" N, 79°57'41.05''E |
| SYSb007964 | *R. d. diluta* | 6.2 | 2.1 | 3.5 | 110 | 82 | 56 | 6.8 | 10.6 | 43°54'43.9"N, 81°14'8.5"E |
| SYSb007965 | *R. d. diluta* | 5.8 | 2.2 | 3.55 | 104 | 79 | 44 | 8 | 10.2 | 43°54'43.9"N, 81°14'8.5"E |
| SYSb007966 | *R. d. diluta* | 5.9 | 2.1 | 3.7 | 108 | 77 | 50.5 | 7.05 | 10.8 | 43°54'43.9"N, 81°14'8.5"E |
| SYSb007967 | *R. d. diluta* | 6.2 | 2.05 | 3.55 | 104 | 81 | 47 | 7.2 | 10.8 | 43°54'43.9"N, 81°14'8.5"E |
| SYSb007969 | *R. d. diluta* | 6 | 2.2 | 4.4 | 102 | 76.5 | 46 | 5.3 | 10.2 | 43°54'43.9"N, 81°14'8.5"E |
| SYSb008009 | *R. d. diluta* | 6.8 | 2.1 | 3.4 | 107 | 82 | 51 | 6.45 | 10.5 | 43°54'43.9"N, 81°14'8.5"E |
| SYSb008010 | *R. d. diluta* | 5.5 | 2 | 3.5 | 108 | 83 | 49 | 5.5 | 10.7 | 43°54'43.9"N, 81°14'8.5"E |
| SYSb008011 | *R. d. diluta* | 5.6 | 1.9 | 3.8 | 100 | 75 | 47 | 5.95 | 10.5 | 43°54'43.9"N, 81°14'8.5"E |
| SYSb008012 | *R. d. diluta* | 5.7 | 1.9 | 3.45 | 106 | 81.5 | 52 | 8.95 | 10.7 | 43°54'43.9"N, 81°14'8.5"E |
| SYSb008334 | *R. d. diluta* | 5.7 | 2 | 4.2 | 106 | 79 | 51 | 7 | 10.9 | 43°54'43.9"N, 81°14'8.5"E |
| SYSb008335 | *R. d. diluta* | 6.4 | 2.05 | 3.3 | 102 | 77.5 | 49 | 6.1 | 10.1 | 43°54'43.9"N, 81°14'8.5"E |
| SYSb008336 | *R. d. diluta* | 6.2 | 2.05 | 3.4 | 104.5 | 81 | 46.5 | 6.7 | 10.3 | 43°54'43.9"N, 81°14'8.5"E |
| SYSb008337 | *R. d. diluta* | 5.2 | 1.9 | 3.3 | 104 | 79.5 | 52.5 | 9.4 | 10.9 | 43°54'43.9"N, 81°14'8.5"E |
| SYSb008338 | *R. d. diluta* | 6.2 | 2 | 3 | 106 | 81.5 | 48 | 5.8 | 10.7 | 43°54'43.9"N, 81°14'8.5"E |
| SYSb008339 | *R. d. diluta* | 6.1 | 1.9 | 3.7 | 109 | 83 | 51 | 7.1 | 10.9 | 43°54'43.9"N, 81°14'8.5"E |
| SYSb008340 | *R. d. diluta* | 6 | 2.1 | 3.9 | 107 | 84 | 52 | 6.1 | 10.7 | 43°54'43.9"N, 81°14'8.5"E |
| SYSb008341 | *R. d. diluta* | 6.1 | 2.1 | 3.5 | 104 | 80 | 51 | 6.2 | 11.5 | 43°54'43.9"N, 81°14'8.5"E |
| SYSb008342 | *R. d. diluta* | 5.2 | 2.1 | 3.4 | 104.5 | 80.5 | 52.5 | 9.3 | 10.8 | 43°54'43.9"N, 81°14'8.5"E |
| SYSb008343 | *R. d. diluta* | 5.5 | 2 | 2.9 | 105 | 81 | 47 | 6.5 | 10.8 | 43°54'43.9"N, 81°14'8.5"E |
| SYSb008344 | *R. d. diluta* | 3.7 | 1.9 | 3.4 | 107 | 82 | 53.5 | 8.7 | 10.9 | 43°54'43.9"N, 81°14'8.5"E |
| SYSb008370 | *R. d. fohkienensis* | 5.55 | 2.23 | 3.14 | 99.3 | 78.5 | 48.2 | 2.55 | 10.79 | 28°48'45.90"N, 104°57'1.65"E |
| SYSb008371 | *R. d. fohkienensis* | 5.58 | 2.22 | 3.83 | 100 | 75.5 | 43.5 | 4.01 | 11.32 | 28°48'45.90"N, 104°57'1.65"E |
| SYSb008372 | *R. d. fohkienensis* | 5.7 | 2.17 | 3.67 | 99.2 | 77.8 | 45.5 | 2.3 | 11.29 | 28°48'45.90"N, 104°57'1.65"E |
| SYSb008373 | *R. d. fohkienensis* | 5.46 | 1.95 | 3.46 | 100 | 76 | 42.8 | 3.16 | 12.5 | 28°48'45.90"N, 104°57'1.65"E |
| SYSb008374 | *R. d. fohkienensis* | 5.83 | 2.17 | 3.24 | 94.5 | 72.5 | 46 | 2.99 | 10.84 | 28°48'45.90"N, 104°57'1.65"E |
| SYSb008375 | *R. d. fohkienensis* | 5.77 | 2.17 | 3.8 | 96 | 76 | 45 | 2.3 | 10.71 | 28°48'45.90"N, 104°57'1.65"E |
| SYSb008376 | *R. d. fohkienensis* | 5.88 | 2.33 | 3.19 | 96 | 74 | 44 | 2.34 | 10.82 | 28°48'45.90"N, 104°57'1.65"E |
| SYSb008377 | *R. d. fohkienensis* | 5.9 | 1.93 | 3.5 | 98 | 78 | 43 | 3.1 | 10.7 | 28°48'45.90"N, 104°57'1.65"E |
| SYSb008378 | *R. d. fohkienensis* | 5.8 | 2.06 | 3.69 | 100 | 76.5 | 47 | 2.1 | 11.13 | 28°48'45.90"N, 104°57'1.65"E |
| SYSb008379 | *R. d. fohkienensis* | 5.45 | 2.05 | 3.23 | 98 | 75 | 47 | 3.23 | 10.59 | 28°48'45.90"N, 104°57'1.65"E |
| SYSb008380 | *R. d. fohkienensis* | 5.36 | 1.93 | 3.3 | 95 | 76 | 45 | 3.49 | 10.94 | 28°48'45.90"N, 104°57'1.65"E |
| SYSb008381 | *R. d. fohkienensis* | 5.84 | 2.15 | 3.5 | 94 | 75.5 | 47 | 3.48 | 10.98 | 28°48'45.90"N, 104°57'1.65"E |
| SYSb008382 | *R. d. fohkienensis* | 5.61 | 2.1 | 3.49 | 100 | 76 | 47.5 | 2.9 | 10.21 | 28°48'45.90"N, 104°57'1.65"E |
| SYSb008383 | *R. d. fohkienensis* | 5.44 | 2.06 | 3.24 | 97 | 74 | 43 | 3.49 | 11.27 | 28°48'45.90"N, 104°57'1.65"E |
| SYSb008384 | *R. d. fohkienensis* | 5.84 | 2.13 | 3.49 | 100 | 78 | 47 | 3.88 | 11.49 | 28°48'45.90"N, 104°57'1.65"E |
| SYSb008385 | *R. d. fohkienensis* | 5.6 | 2.14 | 3.34 | 96 | 76 | 46 | 2.53 | 11.19 | 28°48'45.90"N, 104°57'1.65"E |
| SYSb008386 | *R. d. fohkienensis* | 5.83 | 2.15 | 3.26 | 99.5 | 79 | 47 | 3.93 | 11.93 | 28°48'45.90"N, 104°57'1.65"E |
| SYSb008387 | *R. d. fohkienensis* | 5.43 | 1.94 | 3.1 | 93 | 75.5 | 43 | 1.39 | 11.01 | 28°48'45.90"N, 104°57'1.65"E |
| SYSb008388 | *R. d. fohkienensis* | 5.81 | 2.23 | 3.01 | 95 | 76 | 43.5 | 3.13 | 10.7 | 28°48'45.90"N, 104°57'1.65"E |
| SYSb008389 | *R. d. fohkienensis* | 5.4 | 2.01 | 3.21 | 90 | 69.5 | 41 | 1.95 | 10.51 | 28°48'45.90"N, 104°57'1.65"E |
| SYSb008390 | *R. d. fohkienensis* | 5.72 | 2.25 | 3.36 | 104 | 78 | 47 | 2.51 | 11.15 | 28°48'45.90"N, 104°57'1.65"E |
| SYSb008391 | *R. d. fohkienensis* | 5.92 | 2.03 | 3.21 | 96 | 77 | 46 | 3.54 | 12.35 | 28°48'45.90"N, 104°57'1.65"E |
| NMBE1078288 | *R. d. tibetana* | 5.7 | 2.8 | 3.5 | 110 | 86 | 56 | 5.6 | 10.3 | 47°52.4902'N, 105°47.7577'E |
| NMBE1078289 | *R. d. tibetana* | 5.2 | 2.1 | 3.1 | 103.5 | 82 | 47 | 3.1 | 10.9 | 47°52.4902'N, 105°47.7577'E |
| NMBE1078290 | *R. d. tibetana* | 5.8 | 2.8 | 3.9 | 110 | 86 | 55 | 6.3 | 11.6 | 47°52.4902'N, 105°47.7577'E |
| NMBE1078291 | *R. d. tibetana* | 5.9 | 2.4 | 4.7 | 109 | 85 | 55 | 5.4 | 11.3 | 47°52.4902'N, 105°47.7577'E |
| NMBE1078292 | *R. d. tibetana* | 6 | 2.2 | 3.5 | 109.5 | 80 | 50 | 5.5 | 10.2 | 47°52.4902'N, 105°47.7577'E |
| NMBE1078293 | *R. d. tibetana* | 5.7 | 2.3 | 4 | 101.5 | 79 | 48 | 4 | 10.8 | 47°52.4902'N, 105°47.7577'E |
| NMBE1078297 | *R. d. tibetana* | 5.9 | 2 | 3.8 | 106 | 83 | 44 | 6 | 11.8 | 47°21.5352'N, 103°41.9692'E |
| NMBE1078298 | *R. d. tibetana* | 6.1 | 2.5 | 4.6 | 103.5 | 83 | 55.5 | 6.2 | 9.6 | 47°21.5352'N, 103°41.9692'E |
| NMBE1078299 | *R. d. tibetana* | 5.6 | 2.6 | 3.7 | 109 | 83 | 45 | 3.2 | 10 | 47°21.5352'N, 103°41.9692'E |
| NMBE1078300 | *R. d. tibetana* | 5.7 | 2.3 | 4.5 | 105 | 82 | 45 | 8.2 | 11 | 47°21.5352'N, 103°41.9692'E |
| NMBE1078301 | *R. d. tibetana* | 5.6 | 2.5 | 4.3 | 104 | 79.5 | 46.5 | 6.5 | 11.8 | 47°21.5352'N, 103°41.9692'E |
| NMBE1078302 | *R. d. tibetana* | 4.9 | 2.9 | 4.1 | 108 | 84 | 51.5 | 5.4 | 11.9 | 47°21.5352'N, 103°41.9692'E |
| NMBE1078303 | *R. d. tibetana* | 6.3 | 2.3 | 3.9 | 105 | 84 | 51 | 9.9 | 12.2 | 47°21.5352'N, 103°41.9692'E |
| NMBE1078304 | *R. d. tibetana* | 6 | 2.6 | 3.9 | 105 | 82.5 | 50 | 7.9 | 11.3 | 47°21.5352'N, 103°41.9692'E |
| NMBE1078305 | *R. d. tibetana* | 5.5 | 3 | 3.8 | 106 | 82 | 48 | 6.8 | 11.2 | 47°21.5352'N, 103°41.9692'E |
| NMBE1078306 | *R. d. tibetana* | 5.6 | 3 | 4.6 | 108 | 84.5 | 53 | 7 | 11.5 | 47°21.5352'N, 103°41.9692'E |
| NMBE1078307 | *R. d. tibetana* | 6.7 | 3 | 4 | 110 | 86 | 52 | 5.5 | 11.6 | 47°21.5352'N, 103°41.9692'E |
| NMBE1078287 | *R. d. tibetana* | 5.7 | 3.1 | 3.4 | 105 | 84 | 53 | 8.1 | 10.3 | 47°21.5352'N, 103°41.9692'E |
| NMBE1078308 | *R. d. tibetana* | 6 | 2.5 | 4.3 | 106 | 81.5 | 54 | 5 | 11.5 | 47°21.5352'N, 103°41.9692'E |
| NMBE1078309 | *R. d. tibetana* | 6.1 | 2.7 | 3.8 | 103 | 84 | 44 | 3.9 | 12.3 | 47°21.5352'N, 103°41.9692'E |
| NMBE1078310 | *R. d. tibetana* | 5.7 | 3.1 | 3.9 | 100.5 | 80 | 49.5 | 6.4 | 11.1 | 47°21.5352'N, 103°41.9692'E |

Tale S4 Pairwise F_ST_ (below diagonal) and geographic distances (km, above diagonal) between populations. * indicate significant value p <0.05.

|  | 4 | 5 | 6 | 7 | 8 | 9 | 10 | 11 | 12 | 13 | 14 |
| --- | --- | --- | --- | --- | --- | --- | --- | --- | --- | --- | --- |
| 4 | - | 407 | 657 | 371 | 1908 | 1951 | 1612 | 1501 | 2412 | 3797 | 2486 |
| 5 | 0.04369* | - | 1039 | 754 | 2246 | 2046 | 1749 | 1337 | 2540 | 3912 | 2504 |
| 6 | 0.05371* | 0.00185* | - | 534 | 1270 | 1674 | 1302 | 1689 | 2052 | 3411 | 2295 |
| 7 | 0.03987* | -0.00256 | -0.0031 | - | 1786 | 2110 | 1748 | 1830 | 2531 | 3906 | 2692 |
| 8 | 0.10184* | 0.11141* | 0.11219* | 0.10215* | - | 1460 | 1223 | 2286 | 1499 | 2581 | 2061 |
| 9 | 0.12695* | 0.13138* | 0.1241* | 0.11703* | 0.01405 | - | 372 | 1214 | 512 | 1865 | 651 |
| 10 | 0.15092* | 0.13882* | 0.13465* | 0.1278* | 0.01946* | -0.00199 | - | 1160 | 801 | 2185 | 1012 |
| 11 | 0.14991* | 0.14103* | 0.138* | 0.13131* | 0.01997* | -0.00230 | -0.00651 | - | 1700 | 2905 | 1371 |
| 12 | 0.30626* | 0.34647* | 0.34682* | 0.3293* | 0.25959* | 0.28386* | 0.31289* | 0.31319* | - | 1384 | 663 |
| 13 | 0.32188* | 0.35941* | 0.36299* | 0.34434* | 0.26795* | 0.29988* | 0.32728* | 0.32722* | 0.00637 | - | 1540 |
| 14 | 0.30901* | 0.34877* | 0.34831* | 0.33265* | 0.26096* | 0.28648* | 0.31375* | 0.31441* | 0.00476* | 0.00198* | - |

| **Measurements** | **Comp.1** | **Comp.2** |
| --- | --- | --- |
| Bill to Feathering | 0.215 | -0.005 |
| Bill Depth | 0.269 | -0.413 |
| Bill Width | 0.280 | 0.206 |
| Wing Length | 0.498 | -0.161 |
| P8 (length of third outermost primary) | 0.507 | 0.057 |
| Tail Length | 0.452 | 0.287 |
| Length of Tail Fork | 0.102 | 0.668 |
| Length of Tarsus | 0.288 | -0.478 |

Table S5 Factor loadings of principal component analysis (PCA) based on eight morphometric measurements. Only loadings of the first two components (Comp.1 and Comp.2) are shown.
